# Visual marker–assisted gene editing of *MLO* confers powdery mildew resistance in *Nicotiana alata*

**DOI:** 10.1016/j.abiote.2026.100024

**Published:** 2026-01-18

**Authors:** Hua-Yin Liu, Xin-Yao Huang, Jin-Wang, Yan-Qun Zhang, Jing Li, Ling-Wang Kong, Shi-Ping Zhou, Qiu-Fen Hu, Wei-Guang Wang

**Affiliations:** a Key Laboratory of Natural Products Synthetic Biology of Ethnic Medicinal Resources, State Ethnic Affairs Commission, Yunnan Minzu University, Kunming, 650504, China; b College of Materials and Chemical Engineering, Southwest Forestry University, Kunming, 650224, China; c Yunnan Key Laboratory of Tobacco Chemistry, China Tobacco Yunnan Industrial Co., Ltd., Kunming, 650231, China

**Keywords:** *Nicotiana alata*, Agrobacterium-mediated transformation, Anthocyanin, Powdery mildew, Gene editing

## Abstract

*Nicotiana alata* is a high-value floricultural crop, but its widely cultivated commercial cultivar ‘Jasmine’ is highly susceptible to powdery mildew under low-light indoor conditions. This susceptibility results in leaf mold, yellowing, and wilting, along with a sharp decline in ornamental value. To address this issue and reduce fungicide reliance, we developed the first gene-editing system for *N. alata* featuring antibiotic marker–free visual identification. This system employs dual-visual markers for rapid positive screening using anthocyanin accumulation to indicate successful transformation and albino phenotypes resulting from editing of *NalaPDS* to signal successful gene editing. To enhance the horticultural traits of *N. alata*, we knocked out *NalaMLO*, resulting in T_1_ and T_2_ plants with significantly greater resistance to powdery mildew. Headspace solid-phase microextraction–gas chromatography–mass spectrometry identified 179 floral volatiles, with key odorants such as linalool, phenylethyl alcohol, and d-limonene remaining unaffected in the edited lines. Transcriptomics, metabolomics, and proteomics analyses revealed that the resistance phenotype may be mediated by the coordination of multiple pathways, including the MAPK signaling pathway, starch and sucrose metabolism, fructose and mannose metabolism, and phenylpropanoid biosynthesis. This approach effectively reduces disease-induced losses in the *N. alata* cultivar Jasmine and will accelerate the deployment of disease-resistance traits in ornamental Solanaceae crops.

## 1 Introduction

Flowering tobacco (*Nicotiana alata*) is an ornamental plant species in the Solanaceae family with substantial horticultural value in the global horticultural supply chain, supported by large-scale greenhouse and landscape cultivation [1,2]. Cultivars such as Jasmine are favored for their large, white flowers, extended bloom period, and lily-like or jasmine-like aroma. However, under controlled-environment production and indoor greening conditions, insufficient lighting often results in susceptibility to powdery mildew, causing leaf chlorosis or abscission and a rapid loss of commercial value [3,4]. These production constraints translate into higher input costs, yield loss, and greater fungicide dependence, all issues directly relevant to sustainable agriculture and regulatory compliance.

Modern plant transformation and genome editing technologies enable precise trait deployment, which reduces chemical use and stabilizes quality [5,6]. In practice, however, the utility of these tools hinges on a robust, high efficiency regeneration system that supports reliable transgene delivery, detection, and gene editing. While leaf or hypocotyl regeneration has been reported for *N. alata* [[7], [8], [9]], genotype-dependen poor regeneration limits scalability. Indeed, many varieties exhibit low regeneration efficiency or complete failure to regenerate, restricting large-scale genetic manipulation. Additionally, traditional transformation systems rely on antibiotic resistance markers as well as entail regulatory compliance issues associated with genetically modified organisms (GMOs), which require tedious post-transformation molecular validation and lack real-time visual screening, impeding the high-throughput identification of positive transformants [10,11].

Visual screening systems based on pigment metabolism offer a solution to this problem. For example, genes regulating anthocyanin biosynthesis such as the transcription factor gene *PRODUCTION OF ANTHOCYANIN PIGMENT 1* (*PAP1*) from Arabidopsis (*Arabidopsis thaliana*) and the betalain biosynthesis genes in the RUBY system (including the cytochrome P450 gene *CYP76AD1*, *l**-DOPA 4,5-dioxygenase* [*DODA*], and *Glucosyltransferase* [*GT*]) can induce the accumulation of visible pigments in transformed tissues, enabling the rapid and clear identification of positive transformation events [[12], [13], [14], [15]]. Establishing a visual transgenic system was attempted in *N. alata* using *PAP1*, but these efforts were unsuccessful [9]. This failure might be attributed to the specific white-flowered *N. alata* variety used in this study. In fact, this *N. alata* line is thought to carry a genetic mutation in the anthocyanidin synthase pathway, which would prevent the pigment accumulation used to identify plants successfully transformed with *PAP1*. Thus, overcoming the limitations of cultivar specificity and establishing a universal visual screening system applicable to diverse *N. alata* germplasms are challenges that remain to be met.

The *powdery mildew resistance locus O* (*MLO*) gene family is a key player in plant disease resistance, especially in broad-spectrum resistance to powdery mildew [16,17]. This gene family is highly conserved among different plant species and its members typically encode negative regulators of powdery mildew resistance. Under normal circumstances, the presence of *MLO* inhibits the disease resistance response of plants, and plants with loss-of-function mutations in the *MLO* gene exhibit stronger resistance to powdery mildew. Such mutations have been successfully deployed in various crop species, such as barley (*Hordeum vulgare*) [18], wheat (*Triticum aestivum*) [19], grapevine (*Vitis vinifera*) [20], several cucurbits [21], and tobacco (*N. tabacum*) [22], endowing them with broad-spectrum resistance to powdery mildew. However, the functional characterization of *MLO* homologs and their application in disease-resistant breeding in *N. alata* remains underexplored, partly due to the lack of tools for efficient genetic manipulation of this species.

In this study, we developed an integrated genetic improvement platform for *N. alata* featuring a robust leaf-disc regeneration and transformation system, a visual selection strategy effective across different cultivars with diverse floral colors, and a dual-visual clustered regularly interspaced short palindromic repeats (CRISPR)/CRISPR-associated nuclease 9 (Cas9) editing workflow. We further expanded the editable target range by using the *Streptococcus pyogenes* Cas9 (SpCas9) variants SpG and SpRY for the first time in this species. Using this platform, we generated powdery mildew–resistant lines via targeted knockout of *NalaMLO* without compromising key floral scent components. This approach accelerates breeding efficiency and offers a practical solution for sustainable controlled-environment floriculture.

## 2 Results

### 2.1 Establishment of a stable leaf disc regeneration system and optimization of *Agrobacterium-mediated* transformation in *N. alata*

**Fig. 1:**
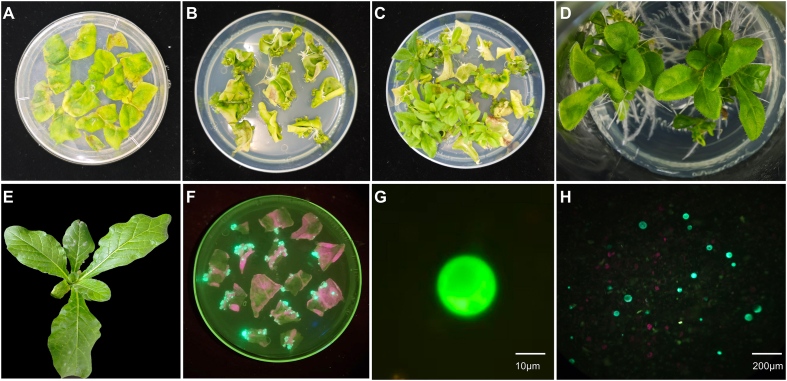
*N. alata* leaf disc regeneration and genetic transformation. **A** Initial leaf disk explants. Callus formation (**B**) and initial shoot differentiation (**C**) from explants. **D** Rooting on MS + 0.1 mg/L NAA. **E** Acclimatized regenerated plants. **F** GFP visualization in transgenic calli. **G** High-magnification GFP fluorescence in a single protoplast. **H** GFP-positive protoplasts from transgenic calli.

Previous attempts to regenerate *N. alata* plants using leaf explants have been inadequately documented or were unsuccessful [8,9]. To establish a reliable regeneration system for *N. alata*, we maintained the 1-naphthaleneacetic acid (NAA) concentration at 0.5 mg/L and tested five concentrations of 6-benzylaminopurine (6-BA; 1, 2, 3, 4, and 5 mg/L) to evaluate their effects on callus induction and shoot differentiation. While all concentrations supported callus formation, higher concentrations of 6-BA (≥4 mg/L) inhibited shoot differentiation and induced callus browning (Fig. 1A−E and Fig. S1). By contrast, 2 mg/L or 3 mg/L 6-BA performed well, achieving >95 % callus induction efficiency and >50 % shoot differentiation efficiency (Fig. S1 and Table S1). Based on these results, we determined the optimal medium composition for callus induction in *N. alata* to be Murashige and Skoog (MS) medium containing 3 mg/L 6-BA and 0.5 mg/L NAA; the optimal combination for bud differentiation induction was MS medium containing 2 mg/L 6-BA and 0.5 mg/L NAA. Finally, for rooting medium, MS medium containing 0.1 mg/L NAA achieved 100 % rooting within 2–4 weeks.

To assess the reliability of the above optimal conditions for genetic transformation, we tested different Agrobacterium (*Agrobacterium tumefaciens*) strains (GV3101, LBA4404, EHA105) and the *A. rhizogenes* strain K599 using a *35S:eGFP* plasmid to infect *N. alata* leaf explants. All strains successfully mediated transformation, as confirmed by GFP fluorescence detected from infected calli and protoplasts isolated therefrom (Fig. 1F–H, Fig. S2). The strains GV3101 and LBA4404 achieved the highest infection efficiencies (∼95 %) with low associated contamination (Table S2, Fig. S3). EHA105 showed similar efficiency but resulted in higher bacterial contamination, likely due to its lower sensitivity to antibiotics. The *A. rhizogenes* strain K599 induced strong GFP fluorescence signal but exhibited high contamination to explants. In addition, K599-infected *N. alata* leaf explants failed to root, making this strain unsuitable for transformation, despite effective rooting induction in *N. tabacum* leaf explants (Fig. S4). This result indicates that the four selected strains can technically all support the genetic transformation of *N. alata*, but there are significant differences in the performance of the different strains.

### 2.2 Establishment of the visual marker–assisted gene editing system in *N. alata*

**Fig. 2:**
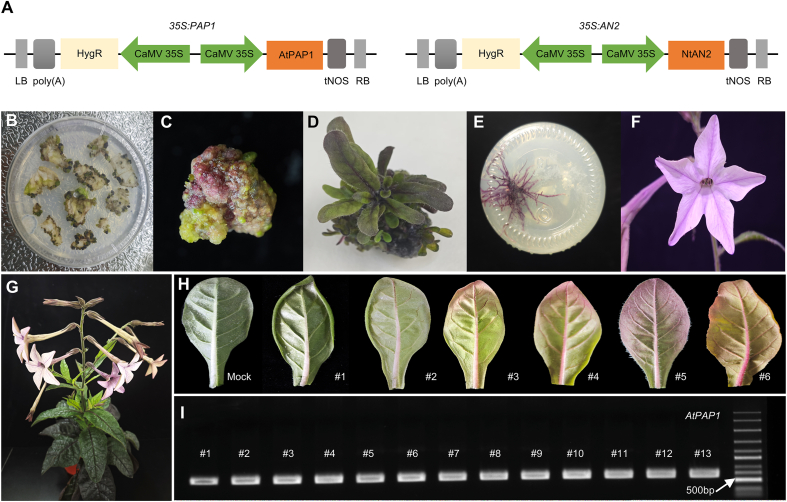
Visual genetic transformation of *N*. *alata* using *PAP1*. **A** Overexpression vectors of constructs with *AtPAP1* and *NtAN*2. **B** Callus induction in transformed explants. **C** Anthocyanin-accumulating callus. **D** Regenerated transgenic shoot with anthocyanin accumulation in tissues. **E** Transgenic roots exhibiting anthocyanin accumulation. **F** Transgenic flower with anthocyanin accumulation. **G** Whole transgenic plant showing anthocyanin distribution in leaves and other tissues. **H** Gradient anthocyanin accumulation in leaves of independent *AtPAP1*-overexpressing lines (#1–#6; Mock: non-transformed control). **I** PCR validation of *AtPAP1* integration in transgenic lines (#1–#13); PCR length: 747 bp.

Previous work reported that *AtPAP1* expression did not yield visible pigment accumulation in a white-flowered line of *N. alata* [9]. To investigate whether genetic differences related to flower color affect *AtPAP1* expression and anthocyanin accumulation, we chose three *N. alata* cultivars with pink, lemon-green, or white flowers for transformation. After constructing the *35S:AtPAP1* vector (Fig. 2A), we conducted *Agrobacterium*-mediated genetic transformation of *N. alata* leaf explants using the strain GV3101; we observed strong anthocyanin accumulation in the callus tissues derived from all three cultivars tested (Fig. 2B, C and Fig. S5). In the white-flowered cultivar, 13 T_0_ plants regenerated from callus displayed purple pigmentation throughout their leaves, vasculature, stems, roots, and flowers (Fig. 2B–G and Fig. S6), with marked variation in anthocyanin accumulation detected across distinct transgenic plants (Fig. 2H). PCR genotyping confirmed the presence of the *35S:AtPAP1* transgene in all tested transgenic plants (Fig. 2I). Additionally, introduction of the *NtAN2* gene [23] from *N. tabacum* into a white-flowered *N. alata* cultivar produced similar anthocyanin accumulation patterns, including purple callus and pigmentation in the stems, leaves, and flowers of regenerated T_0_ plants (Fig. S7). These results indicate that *AtPAP1* and *NtAN2* can both serve as effective visible selection markers in *N. alata* regardless of flower color background.

**Fig. 3:**
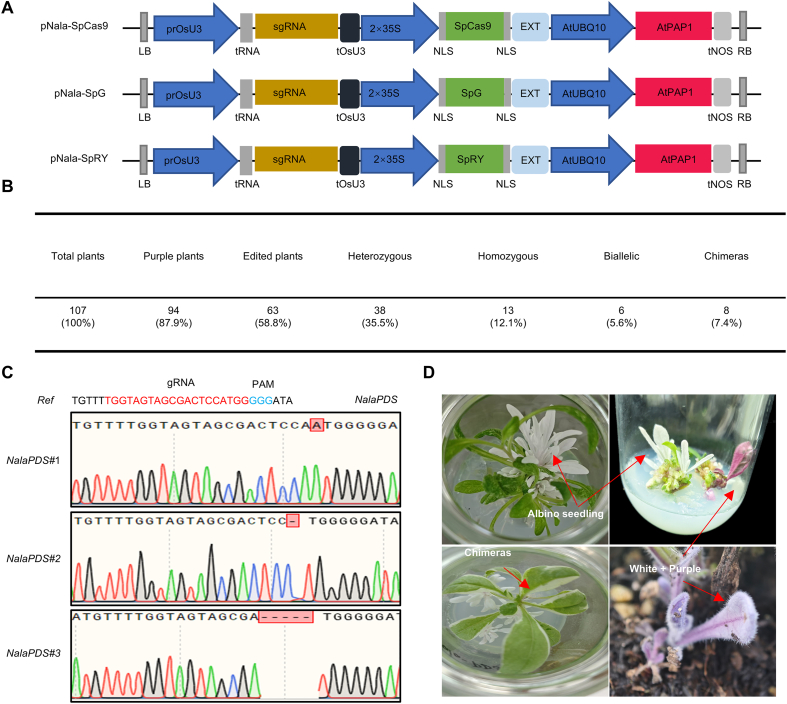
Dual-visualization CRISPR system for *N. alata*. **A** Structures of the constructs pNala-SpCas9 (WT), pNala-SpG (SpG), and pNala-SpRY (SpRY). **B** Statistics of regenerated lines transformed (anthocyanin-positive) lines, and *NalaPDS*-edited genotypes. **C***NalaPDS*-edited lines Sanger sequence and (**D**) phenotype.

Building on the above anthocyanin-based transformation system. we developed three CRISPR/Cas9 vectors for efficient genome editing in *N. alata* with separate visual assessment of transformation status and editing potential (Fig. 3A). The first vector, pNala-SpCas9, consists of (i) an *OsU3* promoter-driven single guide RNA (sgRNA) cassette targeting *Phytoene desaturase* (*NalaPDS*), (ii) an expression cassette encoding nucleus-localized SpCas9, and (iii) an *AtPAP1* expression cassette under the *Arabidopsis* *UBIQUITIN* (*AtUBQ10*) promoter to induce anthocyanin accumulation. This vector supports dual visualization, with anthocyanin accumulation reflecting successful transformation, while the presence of albino tissues caused by editing of *NalaPDS* indicate editing [24], thereby eliminating the need for antibiotic selection. To broaden protospacer adjacent motif (PAM) recognition, we generated two additional vectors pNala-SpG and pNala-SpRY, harboring one of two SpCas9 variants [25]. pNala-SpG carries the variant *SpG*, with its encoded SpG recognizing NGN-type PAMs, while pNala-SpRY harbors *SpRY*, encoding an enzyme capable of near-PAMless (NNN) recognition (Fig. S8). We paired both variants with sgRNA cassettes targeting three endogenous loci (*NalaAN2-like*, *NalaALS-like*, and *NalaPDS*) to evaluate their editing efficiency across different PAM sequences and retained the *35S:AtPAP1* anthocyanin marker cassette.

*Agrobacterium*-mediated transformation with pNala-SpCas9 yielded 107 regenerated primary transformants, of which 94 (87.9 %) exhibited purple pigmentation. Sanger sequencing identified editing events at *NalaPDS* loci in 63 purple plants, corresponding to an overall editing efficiency of 58.8 % (63/107). The genotypes of these edited plants consisted of 38 heterozygous, 13 homozygous, 6 biallelic, and 8 chimeric plants (Fig. 3B). Plants carrying homozygous and biallelic mutations in *NalaPDS* displayed a complete albino phenotype from the cotyledon stage onward while maintaining anthocyanin accumulation in other tissues, producing distinct purple and white phenotypes (Fig. 3C and D). Genotyping PCR confirmed the presence of *SpCas9* in the genome of 13 edited plants (Fig. S9). Chimeric plants were characterized by distinct albino and green sectors, indicating that editing occurred during multicellular regeneration.

**Fig. 4:**
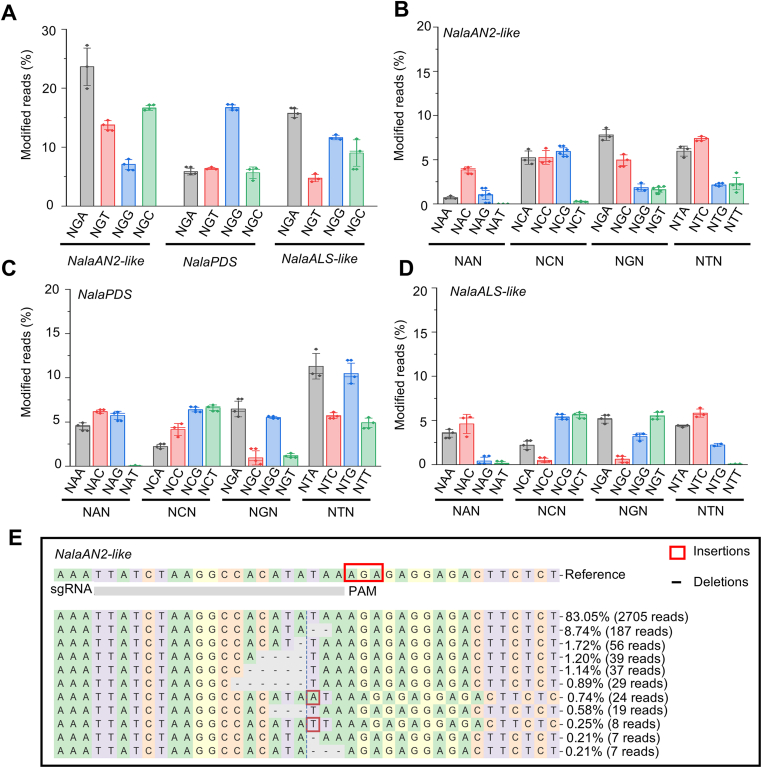
NGS profiling of SpG/SpRY mutation spectra. **A** SpG-mediated mutation frequencies in *NalaAN2-like*, *NalaALS-like*, and *NalaPDS*. **B−D** SpRY-mediated mutation frequencies in *NalaAN2-like*, *NalaALS-like*, and *NalaPDS*. **E** Single-read resolution of SpG-induced mutations at the *NalaAN2-like* target sites.

Amplicon high-throughput sequencing (NGS) of callus tissues transformed with pNala-SpG demonstrated efficient editing across all 12 target sites with NGN-type PAMs tested for the three target genes *NalaAN2-like*, *NalaALS-like*, and *NalaPDS*, with an average editing activity of 11.6 % at target sites with NGG-type PAMs and up to 18.3 % at target sites with NGH-type (H = A, C, or T) PAMs (Fig. 4A). Although lower than the editing efficiency of 37.5 % obtained with SpCas9 at target sites with NGG-type PAMs (Fig. S10), SpG predominantly induced insertions and deletions similar to those generated by SpCas9 (Fig. 4E). Moreover, editing efficiencies varied among target loci, suggesting that the sequence context influences SpG activity. By contrast, transformation with the pNala-SpRY vector exhibited broader PAM compatibility, effectively editing target sites with NRN-type (where R is A or G) PAMs and a subset of target sites with NYN-type (where Y is C or T) PAMs (Fig. 4B–D). However, its overall editing efficiency was lower than that of SpCas9 or SpG. Indeed, SpRY showed lower activity than SpG for 12 target sites with NGN-type PAMs; among 24 target sites with NYN-type PAM sites, 14 were edited with an editing efficiency above 5 %, with a maximum of 11.6 %. These data validate the utility of SpCas9 variants to expand targetable genomic regions in *N. alata* by enabling editing at target sites lacking canonical PAM sites.

The relaxed PAM compatibility of SpRY is often accompanied by self-editing within the sgRNA region of the T-DNA. This phenomenon is commonly observed in both plant and animal cells [26,27]. To assess the self-editing profile of SpRY in *N. alata*, we amplified 14 target loci by PCR and subjected the amplicons to Sanger sequencing. We observed self-editing ranging from 10.0 % to 62.2 % at these sites (Fig. S11). The introduced mutations were mainly insertions and deletions. These findings suggest that although the relaxed PAM requirement of SpRY expands the editable range of genomic regions in *N. alata*, its tendency for self-editing should be carefully considered. This effect may contribute to the overall lower editing efficiency of the system relying on SpRY.

### 2.3 NalaMLO editing confers resistance to powdery mildew

Powdery mildew, caused by *Golovinomyces cichoracearum*, reduces the ornamental value of plants due to the formation of white powdery lesions on their leaves. MLO is a negative regulator of resistance to powdery mildew, and loss-of-function mutations in the encoding gene confer broad-spectrum resistance [16]. In the *N. tabacum* cultivar ‘K326’ (an allotetraploid species, 2n = 4x = 48), simultaneous knockout of *NtMLO1* (derived from *N. tomentosiformis*) and *NtMLO2* (derived from *Nicotiana sylvestris*) confers powdery mildew resistance, confirming these two genes as major resistance genes [22]. Therefore, we speculated that *N. alata* (a diploid species, 2n = 2x = 18) harbors at least one major *MLO* gene that would affect powdery mildew susceptibility. Using BLASTp on transcriptome data, we identified an *MLO* homolog, *NalaMLO*, orthologous to *NtMLO2* (Fig. S12 and Fig. S13).

**Fig. 5:**
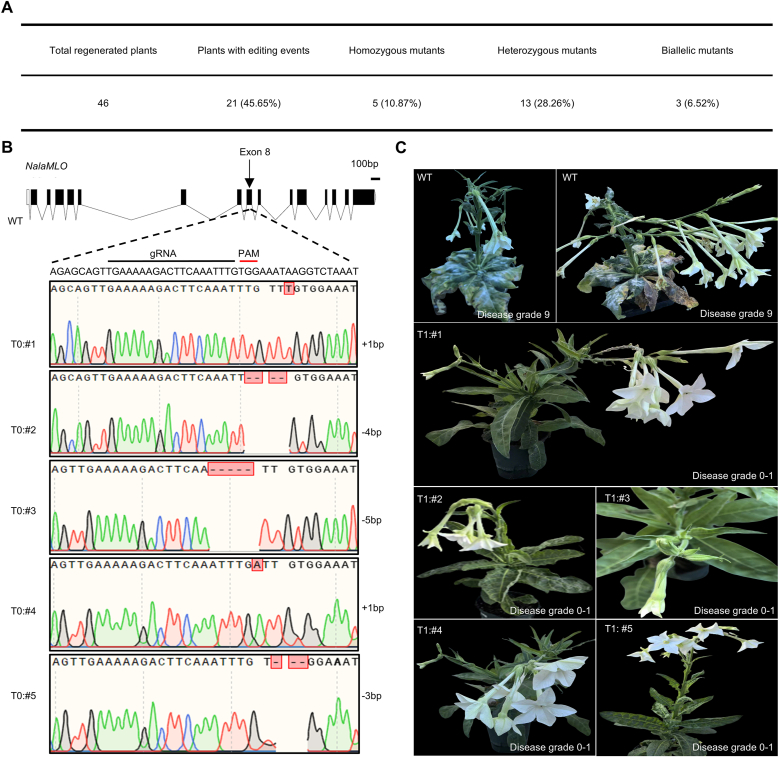
Targeted editing of *NalaMLO* confers powdery mildew resistance in *N. alata*. **A***NalaMLO* gene editing statistic. **B** Sequencing validation of *NalaMLO* gene editing (T_0_). **C** Phenotypic comparison of WT and *NalaMLO*-edited lines (T_1_) two weeks after inoculation with powdery mildew spores (*G. cichoracearum*). WT plants exhibit severe powdery mildew symptoms (disease grade 9), while *NalaMLO*-edited T_1_ lines (#1–#5) show enhanced resistance, with significantly reduced or absent disease symptoms (disease grade 0–1). Grade 0: no visible lesions; Grade 1: lesion area less than 5 % of the leaf area; Grade 9: lesion area greater than 41 % of the leaf area or severe plant wilting.

Due to the abundance of NGG-type PAMs in the *NalaMLO* gene and the high editing efficiency demonstrated by pNala-SpCas9 in *N. alata*, we chose this vector system to perform editing of *NalaMLO* in the self-compatible, susceptible cultivar Jasmine to avoid issues of self-incompatibility. After *Agrobacterium*-mediated leaf disc transformation, we obtained 46 regenerated plants, with 21 (45.6 %) showing edits at *NalaMLO* as confirmed by Sanger sequencing (Fig. 5A). Of these, five plants (#1–#5) were homozygous for small insertion/deletions (InDels) inducing frameshifts primarily in exon 8, while the remaining three plants were biallelic mutants (Fig. 5B), all predicted to disrupt NalaMLO function.

To identify transgene-free *NalaMLO-*edited T_1_ plants, we collected T_1_ seeds from the T_0_ homozygous plant #2 and sowed them in pots. The T_1_ seedlings were either purple or green in appearance (Fig. S14A). Following the method described in previous studies [13], we removed purple seedlings, retaining only green seedlings. Subsequently, we extracted genomic DNA from each remaining green seedling and performed genotyping PCR to detect the T-DNA using specific primers (AtPAP1-F and AtPAP1-R). All green T_1_ plants were free of the transgene (Fig. S14B). We inoculated wild type and these transgene-free T_1_ plants homozygous for mutations in *NalaMLO* with *G. cichoracearum* spores and scored the disease progression after two weeks. While WT plants were highly susceptible (disease grade 9), different T_1_ plants (#1–#5) showed enhanced resistance to powdery mildew (disease grade 0 or 1) and imroved horticultural traits, which were characterized by the absence of visible white mycelial growth or chlorotic lesions on leaves (Fig. 5C). Collectively, these results demonstrate that genome editing of *NalaMLO* confers resistance to powdery mildew in *N. alata* while also optimizing horticultural traits, thereby validating the utility of this approach for improving the agronomic performance of *N. alata*.

### 2.4 Exploring the mechanism behind powdery mildew resistance in *N. alata* conferred by editing of NalaMLO

**Fig. 6:**
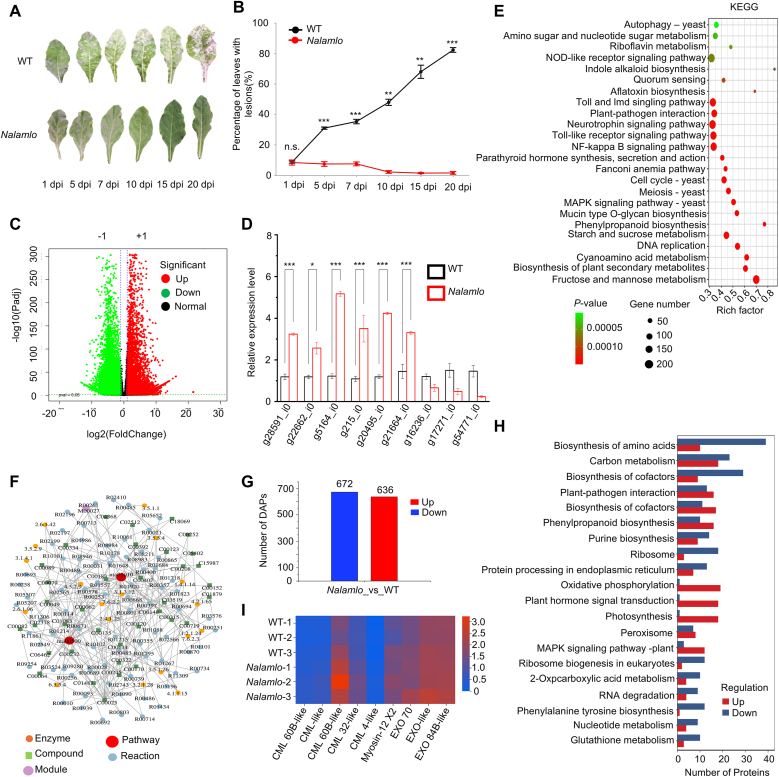
Multi-omics analysis of powdery mildew resistance in *Nalamlo* lines. **A** Phenotypic observation and quantitative assessment of powdery mildew resistance. Left: Representative leaves of WT and *Nalamlo* lines (T_2_) at 1, 5, 7-, 10-, 15-, and 20-days post-inoculation (dpi) with *G. cichoracearum*. **B** Quantification of the proportion of powdery mildew lesion area over time in WT and *Nalamlo* lines (T_2_). **C** Volcano plot of differentially expressed genes (DGEs) between WT and *Nalamlo* lines. Red dots: upregulated genes; green dots: downregulated genes; black dots: non-significant genes. **D** The qPCR validation of DEGs. **E** The KEGG pathway enrichment analysis of DGEs, displaying enriched pathways with corresponding rich factor (*x*-axis), gene count (dot size), and *P*-value (color gradient). **F** Metabolite–enzyme–reaction interaction network. **G** Statistical summary of differentially abundant proteins (DAPs) between *Nalamlo* and WT lines. **H** The KEGG pathway enrichment analysis of DAPs. **I** Heatmap of MLO-interacting proteins. All data are mean ± s.d. Two-tailed Mann–Whitney tests or two-tailed Student's *t*-tests.

We obtained T_2_ generation seeds by selfing green T_1_ plants homozygous for editing of *NalaMLO*. We inoculated these transgene-free homozygous *Nalamlo* lines with *G. cichoracearum* at the seedling stage. We examined leaves at 1, 5, 7, 10, 15, and 20 days post inoculation (dpi). While WT plants showed progressively more severe symptoms over time, the gene-edited *Nalamlo* lines exhibited markedly more limited lesion development (Fig. 6A and B), demonstrating the stable inheritance of resistance to powdery mildew.

To explore the molecular basis of resistance in *N. alata*, we performed transcriptome deep sequencing (RNA-seq) by collecting samples from WT and *Nalamlo* lines at 7 dpi with *G. cichoracearum*. After quality control, 45.56 GB of raw sequencing data were generated. Using |log_2_(fold-change) | > 1.5 and *P*-value <0.01 as criteria, we identified a total of 26,689 differentially expressed genes (DEGs), of which 12,036 were upregulated and 14,653 were downregulated (Fig. 6C). Gene Ontology (GO) term enrichment analysis revealed that the DEGs are predominantly enriched in 42 terms across the categories biological process (BP), cellular component (CC), and molecular function (MF). The enriched BP terms included cellular processes (4190 genes), metabolic processes (3186 genes), bioregulation (2018 genes), and response to stimulus (1661 genes). The enriched CC terms encompassed cell anatomical entity (4522 genes) and protein-containing complex (1488 genes), whereas enriched MF terms included binding (1862 genes), catalytic activity (2519 genes), and transporter activity (351 genes) (Fig. S15). This result suggested that the resistance mechanism of the *Nalamlo* lines involves multiple layers, including signal transduction, metabolic reprogramming, and cellular remodeling (e.g., cell wall fortification, altered plasmodesma permeability, and cytoskeletal rearrangement).

To gather more functional information about these DEGs, we mapped them onto Kyoto Encyclopedia of Genes and Genomes (KEGG) pathways. The DEGs were significantly enriched in pathways associated with stress responses and metabolism, including ‘fructose and mannose metabolism’ (ko00051), ‘starch and sucrose metabolism’ (ko00500), ‘MAPK signaling pathway’ (ko04011), ‘phenylpropanoid biosynthesis’ (ko00940), and the biosynthesis of various plant secondary metabolites (Fig. 6E). These data suggest that *Nalamlo* lines may display enhanced overall immune defense responses through coordinated changes in carbohydrate metabolism, the MAPK signaling pathway, and the phenylpropanoid biosynthesis pathway. We validated the differential expression of nine DEGs from these pathways (six upregulated and three downregulated) using RT-qPCR, obtaining results that were consistent with the RNA-seq data (Fig. 6D).

To investigate the role of specialized metabolites in resistance to powdery mildew, we conducted untargeted metabolomics on WT and *Nalamlo* lines. We identified a total of 640 differentially accumulated metabolites (DAMs), comprising 202 upregulated and 438 downregulated metabolites (Fig. S16A). KEGG pathway enrichment analysis highlighted the significant enrichment of metabolic pathways (Fig. S16B). We focused on top 10 upregulated and downregulated DAMs. The most significantly upregulated metabolites were 2,3,5,7-tetramethoxy-9,10-dihydrophenanthrene; 4-hydroxy-7H-furo[3,2-g]chromen-7-one; veratridine; (E)-2-propenyl-[3-(2-propenylthio)-2-propenyl]-sulfate; spinochalcone C; perseitol; 3′,5′,15′-O-triacetoxy-7′-O-benzoyloxy-14′-O-nicotinate; nortropine; convolicine; and 7-hydroxy-2-phenylchromen-4-one. The top 10 downregulated DAMs were syringic acid; 6-methoxy-7-[(2S,3R,4S,5S,6R)-3,4,5-trihydroxy-6-(hydroxymethyl) tetrahydropyran-2-yl] oxy-2H-chromen-2-one; uridine; *trans*-zeatin; abscisic acid glucose ester; petiline; 4′-methylisoscutellarein-8-(2′-sulfoglucoside); succinate semialdehyde; isorhamnetin-3-glucoside; and prodelphinidin B6 (Fig. S16C). Notably, several of these compounds have known roles in plant defense. For example, spinochalcone C, a flavonoid derivative, exhibits broad-spectrum antibacterial activity by disrupting pathogen cell walls [28], while phenanthrene derivatives, such as 2,3,5,7-tetramethoxy-9,10-dihydrophenanthrene, may function as phytoalexins to inhibit fungal growth [29]. Therefore, in *Nalamlo* lines, the differential abundance of these specific metabolites may be associated with resistance to powdery mildew.

To elucidate the interactions between DAMs and metabolic pathways, we constructed a metabolite–enzyme–reaction network (Fig. 6F). This network revealed that DAMs are predominantly associated with starch and sucrose metabolism (nta00500) and ABC transporters (nta02010), with key metabolites including l-glutamate, l-phenylalanine, sucrose, spermidine, and maltose.

MLO causes susceptibility to powdery mildew by interacting with proteins such as calmodulin (CAM) [18,30], myosin XI [31], and Exocyst Component of 70 kDa (EXO70) [32]. These interactions mainly occur through the cytoplasmic C-terminal domain of MLO and regulate plant susceptibility. To investigate potential changes in protein abundance caused by the loss of MLO function in *Nalamlo* lines, we identified proteins and quantified their levels by liquid chromatography–tandem mass spectrometry (LC-MS/MS). We detected a total of 102,918 unique peptides, corresponding to 13,606 proteins (Fig. S17). Peptide length analysis (Fig. S18) confirmed that 95 % of the peptides within the 95 % confidence interval contain 7–20 amino acids, thus ensuring reliable protein identification. Based on the criteria of a |log_2_(FC)| ≥ 0.585 and a *P*-value ≤0.05, we detected differentially abundant proteins (DAPs), comprising 636 upregulated and 672 downregulated proteins (Fig. 6G). GO term enrichment analysis (Fig. S19) indicated that these DAPs are primarily enriched in terms related to cellular processes, metabolic processes, bioregulation, response to stimulus, and cell anatomical entities, which overlapped with the enrichment results of the DEGs. These observations further corroborate that the loss of NalaMLO function influences signal transduction, metabolic reprogramming, and cellular remodeling through multiple pathways. Additionally, KEGG pathway analysis (Fig. 6H) revealed that both DAPs and DEGs are significantly enriched in the same pathways, such as the MAPK signaling pathway and starch and sucrose metabolism, highlighting the critical roles of these pathways in resistance to powdery mildew. The significantly upregulated DAPs were notably enriched in KEGG pathways related to oxidative phosphorylation (19 proteins), plant hormone signal transduction (18 proteins), and photosynthesis (18 proteins). This finding suggests that *Nalamlo* lines may regulate plant resistance through mechanisms involving oxidative phosphorylation, plant hormone signaling, and photosynthesis.

We observed no significant differences in the expression of *CML* genes, myosin XI genes, or *EXO70*, or in the abundance of their encoded proteins, between WT and *Nalamlo* lines (Fig. 6I). This finding suggests that the mechanism of powdery mildew resistance in *Nalamlo* lines may not be influenced by the levels of these proteins. Furthermore, the potential interactions of these proteins with NalaMLO requires testing.

### 2.5 The effect of gene editing of NalaMLO on the aroma-related components of *N. alata*

**Fig. 7:**
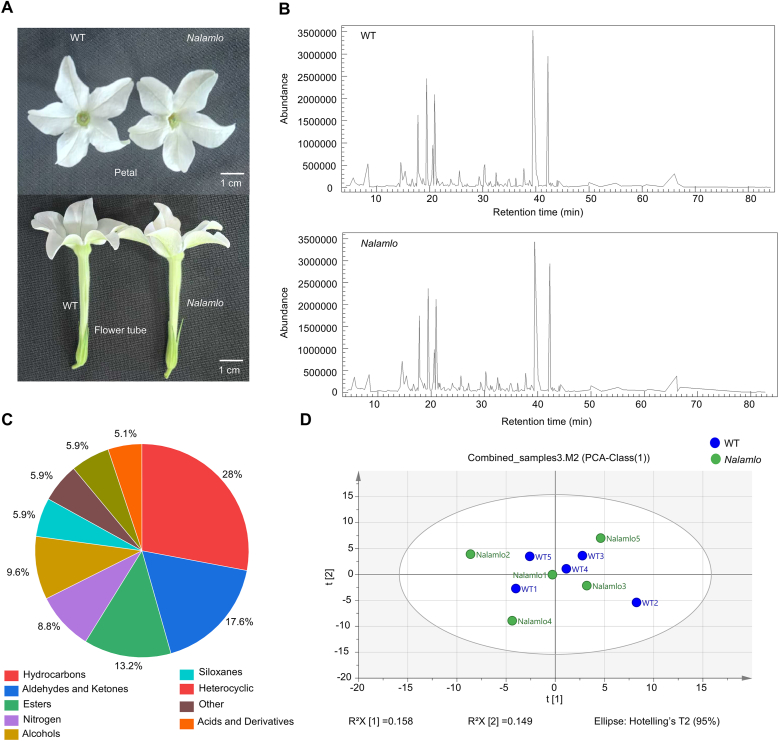
Flower phenotypes and metabolomic profiles of WT and *Nalamlo* lines. **A** Phenotypic comparison of flowers between *Nalamlo* lines and WT. **B** TIC chromatograms of metabolites in WT and *Nalamlo* lines acquired using GC-MS. **C** Pie chart showing the compositional proportions of metabolite classes based on GC-MS analysis, where different colors represent distinct metabolite classes and their respective proportions. **D** PCA score plot of metabolomic data, in which blue dots represent WT samples and green dots represent *Nalamlo* samples.

*N. alata* is an important horticultural crop valued for its floral fragrance and flower color. The loss of MLO function often leads to lower yields and changes in agronomic traits in other crops [33]. To elucidate the consequence of *NalaMLO* gene editing on the ornamental traits of *N. alata*, we analyzed flower size and floral volatile components (VOCs) in WT and *Nalamlo* plants. Phenotypic observations indicated no significant difference in flower size between the WT and *Nalamlo* line (Fig. 7A). For profiling of volatile compounds, headspace solid phase microextraction–GC/MS (HS–SPME–GC/MS) detected over 214 individual compounds, of which 179 were identified. These compounds represent a diverse range of chemical classes, including terpenoids, benzenoids, and fatty acid derivatives (Fig. 7C). Among these identified compounds, 24 compounds have previously been reported as significantly contributing to floral scent (Table 1). For instance, benzaldehyde (14.558 min) is well-known for its characteristic almond-like, sweet aroma and is a common contributor to the pleasant and recognizable scents of many flowers [34]. Linalool (20.924 min) emits a soft floral-citrusy scent and is highly valued for its calming and appealing olfactory properties [35]. This rich blend of compounds gives *N. alata* a unique and appealing floral fragrance.

**Table 1:** List of aroma-related volatile compounds identified in *N. alata* floral tissues via GC–MS analysis.

No.	Retention time (min)	Matching chemical name	Molecular weight (amu)	CAS
1	5.592	Isovaleraldehyde	86.073	590-86-3
2	5.91	Butyl propionate	130.099	590-01-2
3	6.83	2-Heptanol	116.12	543-49-7
4	9.3	2-Methyl-2-pentenal	98.073	623-36-9
5	12.237	2,4-Hexadienal	96.058	142-83-6
6	14.168	2-Heptenal	112.089	57266-86-1
7	14.558	Benzaldehyde	106.042	100-52-7
8	15.151	1-Octen-3-ol	128.12	3391-86-4
9	15.372	5-Hepten-2-one	126.104	110-93-0
10	15.69	2-Pentylfuran	138.104	3777-69-3
11	16.687	2,4-Heptadienal	110.073	4313-03-5
12	17.679	d-Limonene	136.125	5989-27-5
13	17.785	Benzyl alcohol	108.058	100-51-6
14	17.9	3-Octen-2-one	126.104	1669-44-9
15	18.314	Phenylacetaldehyde	120.058	122-78-1
16	20.924	Linalool	154.136	78-70-6
17	21.136	1-Nonanal	142.136	124-19-6
18	21.42	3-Hydroxy-2-methyl-4-pyrone	126.032	118-71-8
19	21.632	Phenylethyl alcohol	122.073	60-12-8
20	22.754	3-Nonen-2-one	140.12	14309-57-0
21	22.99	Lilac aldehyde A	168.115	53447-45-3
22	23.977	Benzyl acetate	150.068	140-11-4
23	25.326	2-Decanone	156.151	693-54-9
24	34.74	*β*-Elemen	204.188	515-13-9

When we compared the volatile profiles of the WT and the *Nalamlo* line, we observed similar peak patterns for the GC/MS total ion current (TIC) chromatograms of WT and *Nalamlo* (Fig. 7B). The retention times and relative peak intensities of the major compounds were nearly identical in the two genotypes, indicating no significant differences in overall volatile compositions. Furthermore, we performed a principal component analysis (PCA) of the volatile components of *Nalamlo* and WT flower samples (Fig. 7D). The first principal component (PC1) explained 15.8 % of the total variance in volatile profiles, while the second principal component (PC2) accounted for 14.9 % of the variance, together capturing approximately 30.7 % of the overall data variability. The points were distributed along a curved trajectory, with the *Nalamlo* and WT samples intermingled without distinct clustering. Overlapping points, such as *Nalamlo* 1 and WT 4 and WT5, suggest a high similarity in chemical profiles. These results indicate that the mutation of *NalaMLO* causes only minor or subtle changes in floral volatiles composition and contents, preserving key metabolic pathways without substantially altering the scent profile.

### 2.6 Nalamlo agronomic traits

**Fig. 8:**
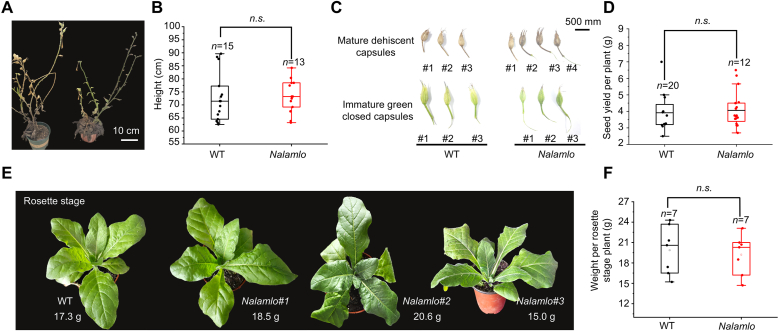
Phenotypic and agronomic trait characterization of *Nalamlo* mutant plants in *N. alata*. **A** Representative plant height phenotype of WT and the *Nalamlo* mutant at the maturing stage. **C** Fruit capsule morphology of WT and *Nalamlo*: mature dehiscent capsules and immature green closed capsules of the two genotypes. **B**, **D**, **F** Box plots of agronomic trait comparisons between WT and *napalm*: (**B**) plant height, (**D**) seed yield per plant, and (**F**) rosette-stage plant weight. “*n.s.*” indicates no significant difference (*P* > 0.05) between genotypes. **E** Representative rosette-stage phenotypes of WT and three independent *Nalamlo* mutant lines (#1, #2, #3), with corresponding fresh weights labeled beneath each plant.

Based on the above findings that editing *NalaMLO* did not affect flower size or floral volatile profiles in *N. alata*, we examined key agronomic traits. We observed no noticeable differences in the appearance of mature WT and *Nalamlo* plants or at the rosette stage (Fig. 8A–E). Capsule development, including both mature dehiscent and immature green capsules, was similar in size and form in the two genotypes (Fig. 8C). Plant height (Fig. 8A and B), seed yield per plant (Fig. 8D), and rosette stage fresh weight (Fig. 8E and F) were also comparable between the two genotypes, confirming that editing *NalaMLO* did not negatively affect these traits. These results are consistent with those of previous reports in the *N. tabacum* cultivar ‘Kokubu’ [36], tomato (*Solanum lycopersicum*) [37], and pea (*Pisum sativum*) [38], in which mutation of *MLO* had little effect on agronomic traits. However, it is worth noting that editing of *MLO* has been linked to lower yield in wheat, suggesting that the roles of *MLO* genes may vary among species.

## 3 Discussion

*N. alata* is an ornamental crop with high horticultural value, and it is also a classic model species for studying plant self-incompatibility [1,39]. However, the genetic improvement of *N. alata* has been plagued by genotype-dependent recalcitrance during regeneration [[7], [8], [9]], limiting the in-depth exploration of this model plant and hindering trait improvement in its commercial cultivars. In this study, we established a stable leaf disc regeneration and *Agrobacterium*-mediated transformation system for *N. alata*, addressing the previous lack of efficient and well-documented protocols for this species. We achieved a transformation efficiency of over 90 % by optimizing the leaf disc regeneration system and *Agrobacterium*-mediated transformation conditions (Fig. S1 and Table S1). The high efficiency observed here in the *N. alata* cultivar Jasmine contrasts with the low efficiency of other genotypes reported previously [8,9], reinforcing that regeneration and transformation success in *N. alata* is strongly genotype-specific.

Of particular importance is the antibiotic-free dual-visual gene-editing system developed in this study, which revolutionizes traditional screening procedures that rely on molecular detection and clarifies a key misunderstanding in the field. Earlier studies failed when using *AtPAP1* as a visual marker in white-flowered *N. alata*, possibly due to mutations in the anthocyanidin biosynthetic pathway in the white-flowered cultivar [9]. However, our study showed stable anthocyanin accumulation induced by *AtPAP1* in *N. alata* cultivars with three flower colors (white, pink, lemon-green). This result demonstrates that the earlier failures are instead probably due to genetic defects in the specific genotype used, and not a universal trait of white-flowered *N. alata*. Our findings provide a unified and intuitive visual marker for genetic screening in cultivars with different flower colors. This system avoids the need to repeatedly adjust the markers for different lines and broadens the use of visual markers in ornamental crops, greatly enhancing their versatility and practicality.

This study is the first to introduce two Cas9 variants into *N. alata*, namely SpG and SpRY, effectively breaking the dependence of traditional SpCas9 on NGG-type PAMs. SpG can efficiently recognize and edit sites with NGN-type PAM with an average editing efficiency of 11.6 %, whereas SpRY can also target sites with non-canonical NRN-type and partial NYN-type PAMs (Fig. 4). The use of SpG and SpRY successfully expands the editable range of the *N. alata* genome, enabling the editing of functional genes containing non-optimal PAMs. Notably, the editing efficiencies of SpG and SpRY are significantly lower than that obtained with SpCas9. Moreover, owing to the relaxed constraints on PAM recognition, both variants have a stronger dependence on the pairing of the sgRNA seed region (3–8 bp upstream of the PAM) [40], which may increase the risk of off-target effects. As *N. alata* currently lacks a high-quality reference genome, we did not systematically test for off-target effects by SpG or SpRY. Indeed, the lack of a reference genome limits the comprehensive evaluation of editing accuracy and poses potential risks to subsequent commercial applications. In the future, priority should be given to completing a genome assembly for *N. alata* cultivar Jasmine to fill this gap.

In this study, targeted editing of *NalaMLO* conferred durable powdery mildew resistance to the cultivar Jasmine. After inoculation with *G. cichoracearum*, homozygous mutants in the T_1_ and T_2_ generations developed significantly smaller leaf lesions and showed no typical disease symptoms, such as chlorosis or wilting, confirming the stable inheritance of resistance. Multi-omics analysis revealed that this resistance is linked to coordinated changes in several pathways, including the activation of the MAPK signaling pathway, remodeling of starch and sucrose metabolism, and regulation of phenylpropanoid biosynthesis. Importantly, HS–SPME–GC–MS detected 179 floral volatiles in the edited plants, with the abundance of key aroma compounds such as linalool, phenylethyl alcohol, and d-limonene remaining unchanged relative to WT. Flower size and other ornamental traits were also stable, demonstrating that enhanced resistance to powdery mildew can be achieved in *N. alata* without compromising its important horticultural traits.

However, some limitations of this study should be noted. Multi-omics data reflect correlations rather than direct causal relationships, as functional validation of core MAPK kinases and antifungal metabolites was not performed. Moreover, all transcriptomic, metabolomic, and proteomic analyses were conducted at a single time point after fungal inoculation (7 dpi), thus precluding the examination of resistance response dynamics. This sampling strategy may have missed some critical information related to earlier or later stages of infection. Future studies should include multiple time points and targeted functional assays to clarify the causal links in NalaMLO-mediated resistance.

In summary, this study integrates an efficient regeneration system, optimized *Agrobacterium*-mediated transformation, universal visualization markers, and PAM-expanded gene editing into a comprehensive genetic improvement technology platform for *N. alata*. This platform enhances powdery mildew resistance while preserving ornamental traits and provides valuable references for developing similar genetic manipulation tools for other ornamental plants and horticultural crops.

## 4 Materials and methods

### 4.1 Plant materials

This study used various *N. alata* accessions featuring pink, lemon-green, or white flowers [41], including the susceptible and self-compatible plastic cultivar 'Jasmine' (commercial variety), to investigate powdery mildew resistance. The plants were cultivated in a greenhouse maintained at 25 ± 2 °C, under a 16-h light/8-h dark photoperiod, with relative humidity of 60–70 %. The plants were cultivated in a greenhouse of Yunnan Minzu University, China.

### 4.2 Establishment of a leaf disc regeneration system

*N. alata* seeds were disinfected by a brief rinse in 75 % (v/v) ethanol (30 s), followed by immersion in 1 % (w/v) AgNO_3_ for 10 min. Seeds were rinsed 3–4 times with sterile distilled water between steps and after the final treatment. Surface-sterilized seeds were sown on half-strength Murashige and Skoog (MS) medium (M519, Phyto Technology Laboratories, Lenexa, USA) and maintained at 25 °C under a 16-h light/8-h dark photoperiod. Healthy young 2–4-week-old seedlings served as the source material for explant preparation. Leaf discs (approximately 0.5 × 0.5 cm) excised from 2 to 4 week-old seedlings were used as explants for callus induction. Callus induction was performed on MS medium supplemented with varying concentrations of 6-BA (1–5 mg/L) and a fixed concentration of NAA (0.5 mg/L). Explants were cultured in the dark at 25 °C, and the callus induction rate was assessed after 2–3 weeks. For adventitious shoot differentiation, calli were transferred to MS medium containing the same plant hormone combinations and cultured under a light intensity of 100 μmol m^−2^ s^−1^. The differentiation rate was recorded after 4–5 weeks. Regenerated shoots (2–3 cm in length) were transferred to MS medium supplemented with 0.1 mg/L NAA for rooting. The rooting rate was determined after two weeks. The following formulas were used to calculate the key efficiency indicators during culture: (1) callus induction rate (%) = number of induced calli/total number of explants × 100 %; (2) shoot differentiation rate (%) = number with shoot differentiation/total number of induced calli; (3) rooting rate (%) = number of induced rooting/total number of induced shoots × 100 %.

### 4.3 Optimization of Agrobacterium-mediated transformation

Agrobacterium (*Agrobacterium tumefaciens* or *Agrobacterium rhizogenes*) competent cells stored at −80 °C were thawed at room temperature or in the palm, and placed on ice. To each 100-μL cell aliquot, 1 μL plasmid DNA (200 ng) was added and mixed by gentle tapping. The mixture was incubated sequentially on ice for 5 min, in liquid nitrogen for 3 min, at 37 °C for 3 min, and on ice for 10 min. Next, 400 μL of YEB medium was added, and the mixture was shaken at 28 °C for 2–3 h. Cells were centrifuged at 8000 *g* for 1 min, ∼100 μL of the supernatant was retained, and the pellet was resuspended. The suspension was spread onto YEB plates with 100 μg/mL kanamycin and 25 μg/mL rifampicin and the plates were incubated at 28 °C for 2 days. Single colonies were picked for colony PCR screening using specific primers. Positive colonies were cultured overnight in 5 mL YEB with antibiotics (25 mg/L rifampicin and 100 mg/L kanamycin) at 28 °C and shaking at 220 rpm until the OD_600_ reached 0.8. Then, 400 μL of this culture was spread onto YEB solid medium with the same antibiotics and incubated overnight at 28 °C. Cells were collected from the plates, resuspended in 10 mL MS medium containing 30 g/L sucrose and 100 μM acetosyringone, and OD_600_ was adjusted to 0.6–0.8.

*Agrobacterium*-mediated transformation was performed using the Agrobacterium strains GV3101, LB4404, EHA105, and K599 (WeidiBio, Shanghai, China). All strains were transformed with the binary vector pCAMBIA1300-35S-EGFP (purchased from Beijing Solarbio Science & Technology Co., Ltd.) containing *GFP* driven by the 35S promoter to facilitate the visual tracking of transformed callus. Leaf disc explants (approximately 0.5 × 0.5 cm) excised from 1-month-old *in vitro–*grown seedlings were immersed in an Agrobacterium cell suspension prepared in antibiotic-free liquid MS medium for 10 min while stationary. The explants were then blotted dry and transferred to callus induction medium containing 200 mg/L kanamycin and timentin to suppress residual bacterial growth. The explants were co-cultivated in the dark at 25 °C for 3 d. The transformation efficiency was assessed two weeks later by observing GFP fluorescence using a fluorescence flashlight (GFPfinder-2101, Xipu (Shanghai) Scientific Instruments Co., Ltd.) with excitation at 488 nm and evaluated as the percentage of fluorescent explants relative to the total number of treated explants. The infection rate of leaf discs was evaluated two weeks after inoculation with *Agrobacterium* (contamination rate = [number of contaminated leaf discs/total number of treated leaf discs] × 100 %). Additionally, protoplasts were isolated from the transformed calli using a Tobacco Protoplasts Isolation and Transformation Kit (Coolaber, Beijing), and GFP fluorescence in protoplasts was observed under a fluorescence microscope (DM IL LED, Germany) to confirm transgene expression.

### 4.4 Transcriptome assembly, reference alignment, and gene functional annotation

For transcriptome analysis of powdery mildew resistance in *N. alata* at 7 days post-inoculation (dpi) with *G. cichoracearum*, three biological replicates of samples from WT plants and homozygous *Nalamlo*-edited lines were collected, with groups matched for growth vigor (4–6 leaf stage) and initial disease severity (no visible symptoms before inoculation); the 3rd and 4th true leaves (primary infection sites) were immediately frozen in liquid nitrogen, ground into fine powder using a pre-chilled mortar and pestle, and stored at −80 °C until RNA extraction. Total RNA was extracted using Trizol reagent (Invitrogen, USA) following the manufacturer's protocol, with on-column DNase I digestion (Qiagen, Germany) to remove genomic DNA contamination, and RNA quality was assessed using a NanoDrop 2000 spectrophotometer (Thermo Fisher Scientific, USA) for purity. Sequencing libraries were constructed using the TruSeq Stranded mRNA Library Prep Kit (Illumina, USA) by enriching mRNA with oligo (dT) magnetic beads, fragmenting into 200–300 bp segments, reverse-transcribing into cDNA with random hexamers, and ligating with Illumina adapters; library quality was validated using the Agilent 2100 Bioanalyzer, followed by paired-end sequencing on the Illumina NovaSeq 6000 platform (Illumina, USA) at Tsingke Biotechnology (Kunming, China). Raw fastq-format reads were processed with in-house Perl scripts to filter out low-quality sequences (Phred score <20), adapter-contaminated reads, and those with poly-N content >5 % to generate clean reads, with quality control metrics including Q20, Q30, GC content, and duplication rate (<15 %) calculated to ensure reliability. Trinity software [42] was used for *de novo* assembly of clean reads to generate the initial transcript sequences. To obtain a nonredundant gene set, the longest transcript for each gene was extracted from the assembled transcripts using a custom Python script, resulting in unigenes in FASTA format. The Trinity-assembled transcriptome was designated as the reference sequence (ref) for downstream alignments. Hisat2 software [43] was used to map the clean reads from each sample to this reference. To predict the protein-coding potential of each gene, unigenes were analyzed using CPC2 software [44]. Unigenes with protein-coding potential were further annotated by sequence alignment against the databases Nr, Swiss-Prot, GO, eggNOG/COG, KOG, KEGG, and Pfam using BLAST. Functional annotation information for these unigenes was integrated from the above-mentioned databases. The detailed experimental methods for transcriptome analysis were compiled as SI-1 in Supplementary Information, which can be referred to for more comprehensive operational details.

### 4.5 Gene cloning and vector construction

Total RNA was extracted from the flowers of *Arabidopsis* and *N. tabacum* plants using a FastPure Universal Plant Total RNA Isolation Kit (Vazyme, Nanjing, China), following the manufacturer's protocol. First-strand cDNA was synthesized via reverse transcription using M-MLV reverse transcriptase and oligo(dT) primers. The full-length coding sequences of *PAP1* (GenBank: NM_104541) and *NtAN2* (GenBank: NM_001325518) were amplified by PCR using gene-specific primers (Table S3). The PCR products were gel-purified using Magen HiPure DNA Clean Up Kit (Guangdong, China) and ligated into the pCAMBIA1304 vector (kindly provided by the Institute of Bioscience, Chinese Academy of Tropical Agricultural Sciences), which was digested with *Sfi*I (NEB, MA, USA), downstream of the 35S promoter. The resulting vectors (*35S:AtPAP1* and *35S:NtAN2*) were verified by Sanger sequencing to confirm correct insertion and sequence integrity before transformation. Three editing vectors (pNala-SpCas9, pNala-SpG, and pNala-SpRY) were constructed based on the Cas9-PF vector framework [13], while containing locus-specific modifications. The base vector pNala-SpCas9, synthesized by GenScript (Nanjing, China), included a *OsU3* promoter–driven tRNA-sgRNA cassette, a 2 × 35S promoter expressing *Cas9* cloned in-frame with the sequence for a nuclear localization signal (NLS), an EXT element, an *AtUBQ10* promoter-driven *PAP1*, and the *Nos* terminator, with the key difference from Cas9-PF being the absence of *NtFT* expression elements. For pNala-SpG, the *Cas9* coding sequence was replaced with *SpG*, while retaining all other vector components. pNala-SpRY was derived from pNala-SpG by replacing the *SpG* coding sequence with that of *SpRY*, with all regulatory elements and marker genes remaining identical to the other two vectors. For target specificity, each of the three vectors was linearized by digestion with *Bsa*I (NEB, MA, USA), and sgRNA oligonucleotides were annealed and ligated into the *Bsa*I-digested plasmids to generate Cas9-PAP1-sgRNA, SpG-PAP1-sgRNA, and SpRY-PAP1-sgRNA constructs. All constructs were validated by Sanger sequencing to confirm the correct insertion, orientation, and sequence integrity before use in the transformation experiments.

### 4.6 Molecular analysis of transgenic plants and visual assessment of CRISPR/Cas9-mediated gene editing

Primary transformants were screened for the accumulation of anthocyanins. Specifically, for transgenic plants harboring the *35S:NtAN2* or *35S:AtPAP1* construct, primary screening was conducted by observing the accumulation of purple anthocyanins in tissues such as calli, leaves, or stems, followed by validation by genotyping PCR. Genomic DNA was extracted from the leaves and calli of these putative transgenic plants using a SolPure Plant DNA Kit (Magen, Guangzhou, China), following the manufacturer's instructions. To identify transgenic plants, PCR amplification was performed using gene-specific primers for *NtAN2* or *AtPAP1* to confirm the stable integration of these marker genes into the *N. alata* genome. Additionally, transgenic plants harboring CRISPR constructs were identified using specific primers for *SpCas9*.

To confirm the editing of *NalaPDS*, specific primers flanking the editing site were used for PCR amplification of genomic DNA from callus and leaves of plants transformed with the *Cas9/NalaPDS*, *SpG/NalaPDS*, or *SpRY/NalaPDS* construct. Fragments of the expected size were recovered and subjected to amplicon-based Fast next-generation sequencing (Fast NGS) and Sanger sequencing at Tsingke Biotechnology. SnapGene software was used to align the sequencing results (Sanger sequencing) with the reference *NalaPDS* sequence to analyze the presence of insertions, deletions, or mutations at the target site. For *NalaPDS*, *NalaAN2-like*, and *NalaALS-like*, high-throughput sequencing data were analyzed using CRISPResso2 (https://crispresso.pinellolab.org/). In addition, for the detection of self-editing during pNala-SpRY-mediated editing, T-DNA-specific primers were used to amplify the sgRNA-containing region of the T-DNA, followed by sequencing to identify sequence variations in the sgRNA region. A similar approach was applied for verification of *NalaMLO* editing: specific primers targeting the *NalaMLO* locus were used for PCR amplification, followed by Sanger sequencing, and sequence alignment was performed to identify mutation types and determine homozygous, heterozygous, biallelic, and chimeric genotypes. To acquire transgene-free *NalaMLO*-edited homozygous plants, the following steps were implemented: seeds collected from T_1_ homozygous plants (designated as T_0_:#2) were sown and purple seedlings were removed, while green seedlings were retained. The green seedlings were verified by genotyping PCR using *AtPAP1*-specific primers to confirm the absence of the transgene, thus obtaining transgene-free T_1_ plants. Subsequently, the screened T_1_ plants were self-pollinated to produce T_2_ lines.

### 4.7 Bioinformatics analysis of NalaMLO and analysis of agronomic traits

The *NalaMLO* gene was identified using BLAST search of the *N. alata* transcriptome database with the coding sequences of *NtMLO1* (GenBank: NM_001325509.1) and *NtMLO2* (GenBank: XM_016633095.2) as queries. The open reading frame of *NalaMLO* was predicted using ORF Finder (https://www.ncbi.nlm.nih.gov/orffinder/), and its encoded amino acid sequence was analyzed for conserved domains using the Conserved Domain Database (CDD, https://www.ncbi.nlm.nih.gov/cdd/). For phylogenetic analysis, full-length amino acid sequences of MLO homologs from *N. alata*, *N. tabacum*, and other representative plant species (e.g., wheat, rice, and tomato) were aligned using ClustalW. A neighbor-joining phylogenetic tree was reconstructed with MEGA11 software [45], using 1000 bootstrap replicates to assess branch support. Sequence identity and similarity were calculated using BLASTp. The agronomic characteristics of the *Nalamlo* line, including plant height (mature stage), weight (resetting stage), capsule development and seed weight, were assessed.

### 4.8 Powdery mildew resistance assays

T_1_ generation plants (derived from selfing of T_0_ homozygous *NalaMLO*-edited plants (#2), T_2_ generation plants (derived from selfing of T_1_ plants) and wild-type controls were used for resistance evaluation. *G. cichoracearum* conidia were collected from infected leaves of wild-type *N. alata* plants artificially inoculated with the fungal isolate originally obtained from naturally infected *N. alata* individuals in our experimental greenhouse, resuspended in sterile water containing 0.02 % (v/v) Tween-20, and adjusted to a titer of 3 × 10^5^ conidia/mL. Seedlings at the 4–6 leaf stage were inoculated by spraying the conidial suspension evenly onto the leaf surface until runoff. Inoculated seedlings were maintained in a greenhouse at 22–25 °C with 70–80 % relative humidity and under a 16-h light/8-h dark photoperiod. Disease severity was assessed at 1, 5, 7, 10, 15, and 20 days post-inoculation (dpi) based on the percentage area of symptoms on each plant leaf [46]. Disease grading was performed in accordance with the national standard GB/T23222-2008.

### 4.9 Omics analysis

The detailed experimental methods for metabolomics and proteomics analysis are provided in Supplementary Information as SI-2 and SI-3, respectively.

### 4.10 HS–SPME–GC–MS analysis and identification

A 5977BGC/MSD gas chromatography-mass spectrometer (Agilent, USA) was used to analyze the volatile components of the flower. The headspace solid-phase microextraction (HS–SPME) conditions were as follows: a DVB/Carbon WR/PDMS-120 μm extraction head was used; the extraction temperature was 70 °C; rotating speed was 250 r·min^−1^; the extraction time was 30 min; the desorption time was 2 min. For GC, the chromatographic column was a DB-5MS column (60 m × 0.25 mm, 0.25 μm); the initial column temperature was 60 °C, held for 2.0 min; increased to 300 °C at a rate of 3 °C·min^−1^, held for 8.0 min; injection port temperature was 260 °C; carrier gas was He, and the flow rate was 1.0 mL min^−1^; the split ratio was 5:1. For MS, the temperature of the transmission line was 260 °C; ion source temperature was 230 °C; ionization mode was provided by electron bombardment (EI) as ion source; ionization energy was 70 eV; the detection mode was full scan monitoring mode; the scanning range was 35–450 amu. Compound identification was performed by GC–MS using commercial mass spectral libraries (NIST 14) and retention times (linear retention index, LRI).

### 4.11 Statistical analysis

All experiments were performed in triplicates (*n* ≥ 3 explants/plants per replicate). Data were analyzed using analysis of variance (ANOVA) with Duncan's multiple range test (*P* < 0.05) in SPSS version 22.0. Principal component analysis (PCA) was performed using SIMCA 14 software.

## CRediT authorship contribution statement

**Hua-Yin Liu:** Writing – original draft, Validation, Resources, Investigation. **Xin-Yao Huang:** Validation. **Jin-Wang:** Writing – original draft, Investigation. **Yan-Qun Zhang:** Data curation. **Jing Li:** Resources. **Ling-Wang Kong:** Software. **Shi-Ping Zhou:** Writing – review & editing. **Qiu-Fen Hu:** Supervision, Resources, Project administration, Methodology, Funding acquisition, Formal analysis, Conceptualization. **Wei-Guang Wang:** Supervision, Resources, Project administration, Methodology, Investigation, Funding acquisition, Formal analysis, Conceptualization.

## Data availability

All data supporting the findings of this study are available within the article and supplementary files.

## Declaration of competing interest

The authors declare that they have no known competing financial interests or personal relationships that could have appeared to influence the work reported in this paper.
