# Supplementary material for: Visual marker–assisted gene editing of MLO confers powdery mildew resistance in Nicotiana alata
Source: aBIOTECH. 2026 Jan 18;7(2):100024. doi: 10.1016/j.abiote.2026.100024 (PMC12973411; doi:10.1016/j.abiote.2026.100024)
Supplement: Multimedia component 1 [file mmc1.docx]

**SUPPORTING INFORMATION**

**Visual marker–assisted gene editing of *MLO* confers powdery mildew resistance in *Nicotiana alata***

Hua-Yin Liu^a,b^, Xin-Yao Huang^a^, Jin-Wang^c^, Yan-Qun Zhang^a^, Jing Li^c^, Ling-Wang Kong^a^, Shi-Ping Zhou^b^, Qiu-Fen Hu^a,c,^*^*^*, Wei-Guang Wang^a,^*^*^*

^a^ Key Laboratory of Natural Products Synthetic Biology of Ethnic Medicinal Resources, State Ethnic Affairs Commission, Yunnan Minzu University, Kunming, 650504, China

^b^ College of Materials and Chemical Engineering, Southwest Forestry University, Kunming, 650224, China

^c^ Yunnan Key Laboratory of Tobacco Chemistry, China Tobacco Yunnan Industrial Co., Ltd., Kunming, 650231, China

**Contents of Supporting Information**

**SI-1** Transcriptomic analysis

**SI-2** Metabolomic analysis

**SI-3** Proteomic analysis

**Supplementary figure and table.**

**Table S1.** Culture conditions for *N. alata* tissue culture.

**Table S2.** Efficiency of different *Agrobacterium* strains in mediating genetic transformation of *N. alata* explants.

**Table S3.** Primers for CRISPR-Cas9 vector construction and mutation detection of gene editing in *N. alata*.

**Fig. S1.** The effects of 6-BA concentration on callus induction and shoot regeneration in *N. alata.*

**Fig. S2.** *Agrobacterium*-mediated transformation of leaf-derived callus tissues using different *Agrobacterium* strains (K599, LBA4404, GV3101, EHA105).

**Fig. S3.** The leaf disk contamination rates in different *Agrobacterium* strains within two weeks.

**Fig. S4.** Comparison of *Nicotiana alata* (left) and *Nicotiana tabacum* (right) explants infected with *R. rhizogenes* K599.

**Fig. S5.** Callus phenotypes of *AtPAP1*-transgenic *Nicotiana alata* with different flower colors.

**Fig. S6.** Phenotypes of *N. alata* transgenic lines overexpressing *AtPAP1*.

**Fig. S7.** Phenotypic observation of *NtAN2* transgenic *N. alata.*

**Fig. S8.** PCR verification results of SpCas9 integration in randomly selected *PDS* edited lines of *N. alata*.

**Fig. S9.** Schematic of SpCas9 and its variants SpG and SpRY.

**Fig. S10.** NGS results of SpCas9-mediated editing at the *N. alata* *PDS* gene.

**Fig. S11.** Sequence characteristics and frequency statistics of self-editing events in SpRY-mediated gene editing.

**Fig. S12.** Phylogenetic tree of *NalaMLO* gene.

**Fig. S13.** Multiple sequence alignment of MLO protein sequences from various plant species.

**Fig. S14.** Screening for transgene-free *MLO*-edited T_1_ plants.

**Fig. S15.** Bar chart of Gene Ontology (GO) analysis for transcripts, categorizing terms into Biological Process (BP, blue), Cellular Component (CC, red), and Molecular Function (MF, green).

**Fig. S16.** Metabolomic analysis of differentially accumulated metabolites (DAMs) between wild-type (WT) and *Nalamlo* lines.

**Fig. S17.** Summary of mass spectrometry-based proteomic profiling.

**Fig. S18.** Distribution of peptide length and charge in proteomic analysis.

**Fig. S19.** Gene Ontology (GO) enrichment analysis of differentially abundant proteins (DAPs).

*SI-1 Transcriptomic analysis*

For transcriptome analysis of powdery mildew resistance in *N. alata* at 7 days post-inoculation (dpi) with *G. cichoracearum*, three biological replicates of samples from wild-type (WT) plants and homozygous *Nalamlo*-edited lines were collected, with groups matched for growth vigor (4–6 leaf stage) and initial disease severity (no visible symptoms before inoculation); the 3rd and 4th true leaves (primary infection sites) were immediately frozen in liquid nitrogen, ground into fine powder using a pre-chilled mortar and pestle, and stored at -80 °C until RNA extraction. Total RNA was extracted using Trizol reagent (Invitrogen, USA) following the manufacturer’s protocol, with on-column DNase I digestion (Qiagen, Germany) to remove genomic DNA contamination, and RNA quality was assessed using a NanoDrop 2000 spectrophotometer (Thermo Fisher Scientific, USA) for purity. Sequencing libraries were constructed using the TruSeq Stranded mRNA Library Prep Kit (Illumina, USA) by enriching mRNA with oligo (dT) magnetic beads, fragmenting into 200–300 bp segments, reverse-transcribing into cDNA with random hexamers, and ligating with Illumina adapters; library quality was validated using the Agilent 2100 Bioanalyzer, followed by paired-end sequencing on the Illumina NovaSeq 6000 platform (Illumina, USA) at Tsingke Biotechnology (Kunming, China). Raw fastq-format reads were processed with in-house Perl scripts to filter out low-quality sequences (Phred score < 20), adapter-contaminated reads, and those with poly-N content > 5% to generate clean reads, with quality control metrics including Q20, Q30, GC content, and duplication rate (< 15%) calculated to ensure reliability. Given the lack of a published reference genome for *N. alata*, clean reads from all samples were de novo assembled into a transcriptome using Trinity software (v2.14.0) with default parameters, and the longest transcript for each gene (unigene) was retained; clean reads of each sample were mapped to the assembled unigene reference using Hisat2 software (v2.2.1), with mapping rates ≥ 85% considered acceptable. Gene functional annotation was performed by aligning unigenes against Nr, Swiss-Prot, Pfam, KOG/COG, GO, KO, and Nt databases using BLAST (v2.12.0) with an E-value cutoff of 1e-5. Gene expression levels were quantified as FPKM (fragments per kilobase of transcript per million mapped reads) using RSEM software (v1.3.3), and differential expression analysis between WT and edited lines at 7 dpi was conducted using DESeq2 (v1.34.0) in R, with DEGs (differentially expressed genes) identified by the criteria of FDR < 0.05 and |log_2_(fold change)| ≥ 1.5. GO enrichment analysis of DEGs was performed using the GOseq R package (v1.46.0) (with significantly enriched terms defined by corrected *P*-value < 0.05), and KEGG pathway enrichment analysis was conducted using KOBAS software (v3.0) (with significant pathways defined by corrected *P*-value < 0.05). To validate transcriptome results, nine DEGs (six upregulated and three downregulated) involved in key pathways (e.g., MAPK signaling pathway, phenylpropanoid biosynthesis, starch and sucrose metabolism) were selected for qRT-PCR verification; first-strand cDNA was synthesized using the PrimeScript RT Reagent Kit with gDNA Eraser (TaKaRa, Japan), qRT-PCR was performed on a CFX96 Real-Time PCR Detection System (Bio-Rad, USA) using SYBR Premix Ex Taq II (TaKaRa, Japan), with the actin gene of *N. alata* as the internal reference, and relative expression levels calculated using the 2^^-ΔΔCt^ method. Primer sequences are detailed in Table 3.

*SI-2 Metabolomic analysis*

For metabolomic analysis of powdery mildew resistance in *N. alata* at 7 days post-inoculation (dpi) with *G. cichoracearum*, sample preparation was consistent with that for transcriptomics: three biological replicates of leaf samples (3rd and 4th true leaves) from WT plants and homozygous *NalaMLO*-edited lines were collected, with groups matched for growth vigor (4–6 leaf stage) and initial disease severity (no visible symptoms before inoculation); the collected plant samples (20 ± 1 mg) were lyophilized and homogenized with beads in 1000 μL of MeOH:ACN:H₂O (2:2:1, v/v), then homogenized (35 Hz, 4 min) and sonicated for 5 min in a 4℃ bath, with this step repeated three times, followed by vortexing, re-sonicating, incubating at –40 ℃for protein precipitation, centrifuging, transferring to protein precipitation plates, and vacuum treatment at 6 psi for 120 seconds to obtain supernatants, while quality control (QC) samples were prepared by pooling equal aliquots of supernatants from all samples. Nonpolar metabolites were analyzed using a Vanquish UHPLC system (Vanquish, Thermo Fisher Scientific) coupled to an Orbitrap Exploris 120 MS (Orbitrap MS, Thermo) with a Phenomenex Kinetex C18 column (2.1 mm × 100 mm, 2.6 μm), with mobile phases consisting of 0.01% acetic acid in water and IPA: ACN (1:1, v/v), a column temperature of 25 °C, and the mass spectrometer operated in IDA mode under ESI± conditions with specific settings for sheath gas, aux gas, and spray voltage. Raw data were converted to mzXML and processed with an in-house R-based program leveraging XCMS; after RSD denoising and imputation of missing values, the data were normalized and analyzed using SIMCA18.0.1, with PCA applied for outlier detection, OPLS-DA with 7-fold cross-validation and 200 permutations identifying metabolites with VIP > 1 and *P* < 0.05 as significantly changed, and pathway enrichment analysis conducted using KEGG (http://www.genome.jp/kegg/) and MetaboAnalyst (<http://www.metaboanalyst.ca/>).

*SI-3 proteomic analysis*

Total protein extraction was performed using SDT lysis buffer (4% SDS, 100 mM Tris-HCl, 10 mM DTT, 1 mM PMSF, 2 mM EDTA) followed by boiling at 95 °C for 15 min and ultrasonic disruption on ice for 10 min. After centrifugation at 12,000 × *g* for 15 min, supernatants were subjected to protein precipitation with 4 volumes of cold acetone at -20 °C overnight. Protein pellets were washed three times with pre-chilled acetone, re-dissolved in 8 M urea, and quantified using a BCA protein assay kit (Beyotime) with bovine serum albumin as standard.

For enzymatic digestion, 100 μg of protein from each sample was adjusted to 200 μL with 8 M urea, reduced with 5 mM DTT at 37 °C for 45 min, and alkylated with 11 mM iodoacetamide in the dark at room temperature for 15 min. Trypsin digestion (Promega V5280) was performed overnight at 37 °C at a 1:50 enzyme-to-protein ratio in 25 mM ammonium bicarbonate. Digested peptides were acidified to pH 2–3 with 20% trifluoroacetic acid, desalted using C18 solid-phase extraction columns (Millipore), and quantified using the Pierce™ Quantitative Peptide Assay Kit.

Liquid chromatography-tandem mass spectrometry (LC-MS/MS) analysis was conducted on a Vanquish Neo UHPLC system coupled to an Orbitrap Astral mass spectrometer (Thermo Fisher Scientific). Peptides were separated on an Easy-Spray^TM^ PepMap^TM^ Neo UHPLC column (150 mm × 0.15 mm, 2 μm) with a gradient elution (mobile phase A: 0.1% formic acid in water; mobile phase B: 0.1% formic acid in acetonitrile) at a flow rate of 2.5 μL/min over 22 min, with the column temperature maintained at 55 °C. Data-independent acquisition (DIA) mode was employed for MS detection: primary mass spectra were acquired over m/z 380–980 with a resolution of 240,000 at 200 m/z, followed by MS/MS analysis using 299 consecutive scan windows (2 Th isolation width) with 25% HCD collision energy.

Raw MS data were processed using DIA-NN v1.8.1 with a library-free search strategy against the UniProt *Nicotiana tabacum* database (uniprotkb_proteome_UP000084051). False discovery rates (FDR) were controlled at <1% for both protein and precursor ion levels. Protein quantification was performed using the MaxLFQ algorithm, with normalization to the median intensity of each sample. Differentially expressed proteins (DEPs) were identified using Student’s *t*-test with thresholds set at fold change (FC) ≥ 1.5 or ≤ 0.667 and *P*-value ≤ 0.05.

Bioinformatics analyses included: functional annotation against GO, KEGG, and KOG databases; enrichment analysis using ClusterProfiler (v4.4.4) with hypergeometric testing; protein domain prediction via InterProScan (v5.59-91.0); subcellular localization using WoLF PSORT; signal peptide prediction with SignalP 5.0; protein-protein interaction (PPI) network construction using StringDB (confidence score > 400); and weighted protein co-expression network analysis (WPCNA) implemented via the WGCNA package in R.

**Table S1.** Culture conditions for *N. alata* tissue culture.

| Experimental Steps | Concentration/Proportion | Culture times |
| --- | --- | --- |
| Callus induction | MS + 30 g/L sucrose + 0.8% agar +3 mg/L 6-BA+0.5 mg/L NAA | 2-6 weeks |
| Shoot regeneration | MS + 30 g/L sucrose + 0.8% agar +2 mg/L 6-BA+ 0.5 mg/L NAA | 2-3 weeks |
| Rooting | MS + 30 g/L sucrose + 0.8% agar+0.1 mg/L NAA | 2-4 weeks |

**Table S2.** Efficiency of different *Agrobacterium* strains in mediating genetic transformation of *N. alata* explants.

| Agrobacterium Strain | Vector | No.of Inoculated Explants | No. of Calli Formed | No. of GFP-Positive Calli |
| --- | --- | --- | --- | --- |
| LBA4404 | 35S::GFP | 189 | 184 | 180 (95.2%) |
| GV3101 | 35S::GFP | 203 | 198 | 194 (95.5%) |
| EHA105 | 35S::GFP | 186 | 177 | 170 (91.4%) |
| K599 | 35S::GFP | 208 | 163 | 159 (76.4%) |

| Primer name | Sequence 5' - 3' | Use |
| --- | --- | --- |
| NalaPDS-sg1-F | TGCATGGTAGTAGCGACTCCATGG | NalaPDS gene editing |
| NalaPDS-sg2-F | TGCATTTTTCAGGGTGTGCCTGATA | NalaPDS gene editing |
| NalaMLO-sg1-F | TGCAAAAAGACTTCAAATTTGTTG | NalaMLO gene editing |
| NalaPDS-sg1-R | AAACCCATGGAGTCGCTACTACCA | NalaPDS gene editing |
| NalaPDS-sg2-R | AAACTATCAGGCACACCCTGAAAA | NalaPDS gene editing |
| NalaMLO-sg1-R | AAACCAACAAATTTGAAGTCTTTT | NalaMLO gene editing |
| MLO-sgJC-F1 | ACGTCATTTGGACGTAGG | Target-detecting of the *NalaMLO* gene |
| NalaPDS-JC-F1 | TGGGAACTGAAAGTCAAGATG | Target-detecting of the *NalaPDS* gene |
| NalaPDS-JC-R1 | CTACCTTTAAAGGATTAAAGTCC | Target-detecting of the *NalaPDS* gene |
| NtAN2-KL-F | ATGAATATTTGTACTAATAA | NtAN2 gene cloning and Transgenic verification |
| NtAN2-KL-R | TCAACTGAAAAGTGGCATTTCC | NtAN2 gene cloning and Transgenic verification |
| AtPAP1-KL-F | ATGGAAGGATCTTCAAAAGGTC | AtPAP1 gene cloning and Transgenic verification (T-DNA) |
| AtPAP1-KL-R | TCAATCCAACTCAACAGTTTC | AtPAP1 gene cloning and Transgenic verification (T-DNA) |
| NtAN2-EP-F | TCTCTGTCCAGTCCTGGCCTATGAATATTTGTACTAATAA | 35:AN2 vector construction |
| NtAN2-EP-R | TCAACTGAAAAGTGGCATTTCCCAGCAGACCACAAGTGGCCA | 35:AN2 vector construction |
| AtPAP1-EP-F | TCTCTGTCCAGTCCTGGCCTATGGAAGGATCTTCAAAAGGTC | 35:PAP1 vector construction |
| AtPAP1-EP-R | TCAATCCAACTCAACAGTTTCCAGCAGACCACAAGTGGCCA | 35:PAP1 vector construction |
| Cas9-F | TACACCAGACGGAAGAACCG | Transgenic verification SpCas9 |
| Cas9-R | GTCCAGGAAGTCCTTGTCCTT | Transgenic verification SpCas9 |
| MLO-sgJC-R1 | TCCACAACAAATTTGAAGTCTT | Target-detecting of the *NalaMLO* gene |
| SPRY-NGS-F1 | TCGTCGGCAGCGTCAGATGTGTATAAGAGACAGGCAGATGATCCGTGGCAACA | Self-editing |
| SPRY-NGS-R1 | GTCTCGTGGGCTCGGAGATGTGTATAAGAGACAGGCGACGGAGAAAACTCAATG | Self-editing |

**Table S3.** Primers for CRISPR-Cas9 vector construction and mutation detection of gene editing in *N. alata*.


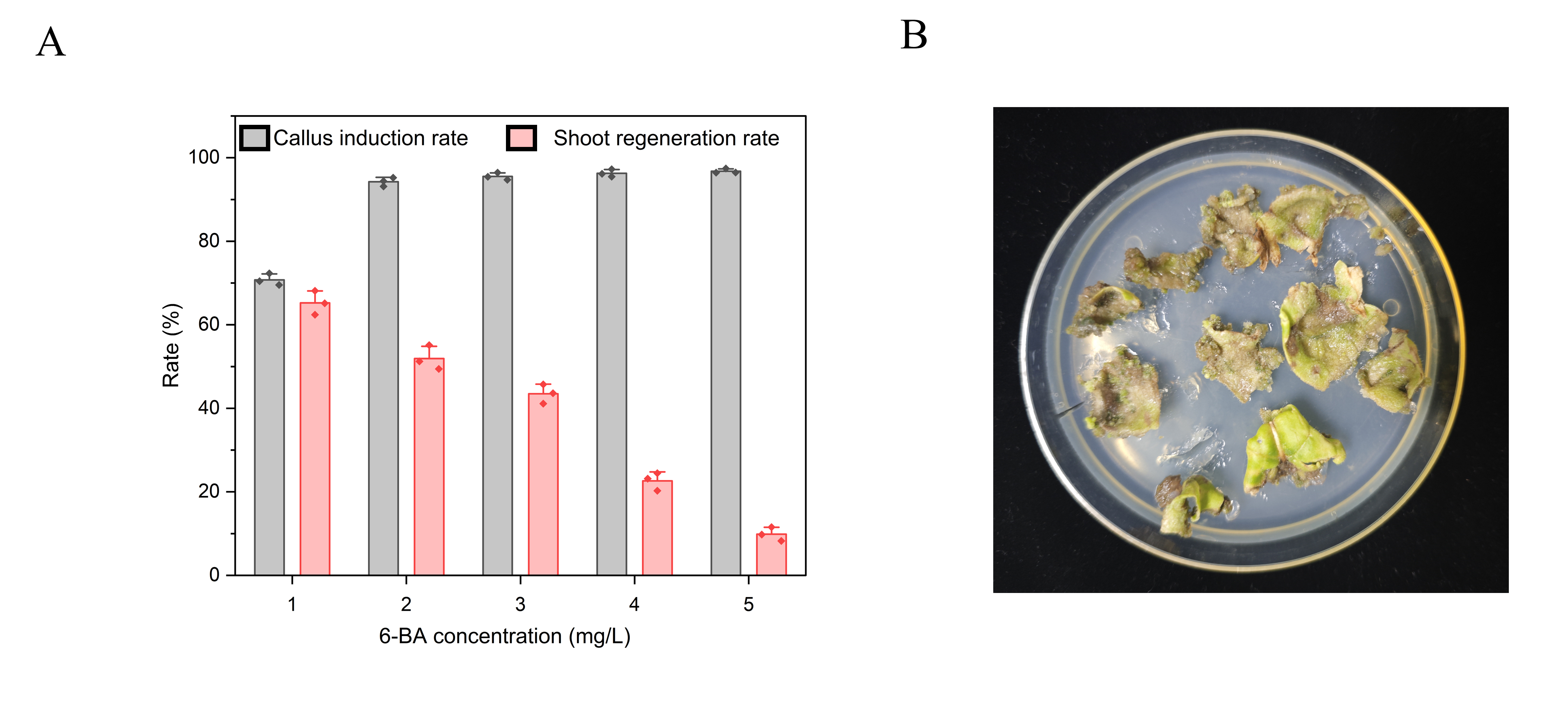


**Fig. S1** The effects of 6-BA concentration on callus induction and shoot regeneration in *N. alata*. **A** Callus induction and shoot regeneration rates of *N. alata* under varying 6-BA concentrations. **B** Callus growth state of *N. alata* under 5 mg/L 6-BA treatment.


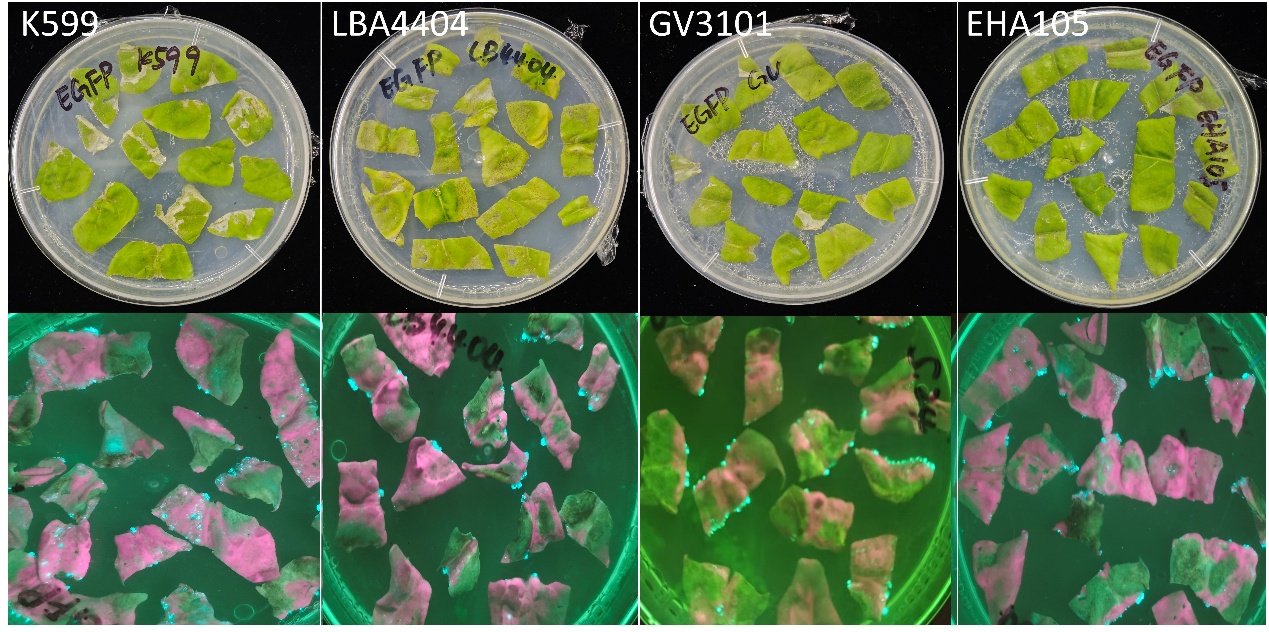


**Fig. S2** *Agrobacterium*-mediated transformation of leaf-derived callus tissues using different *Agrobacterium* strains (K599, LBA4404, GV3101 and EHA105).

**
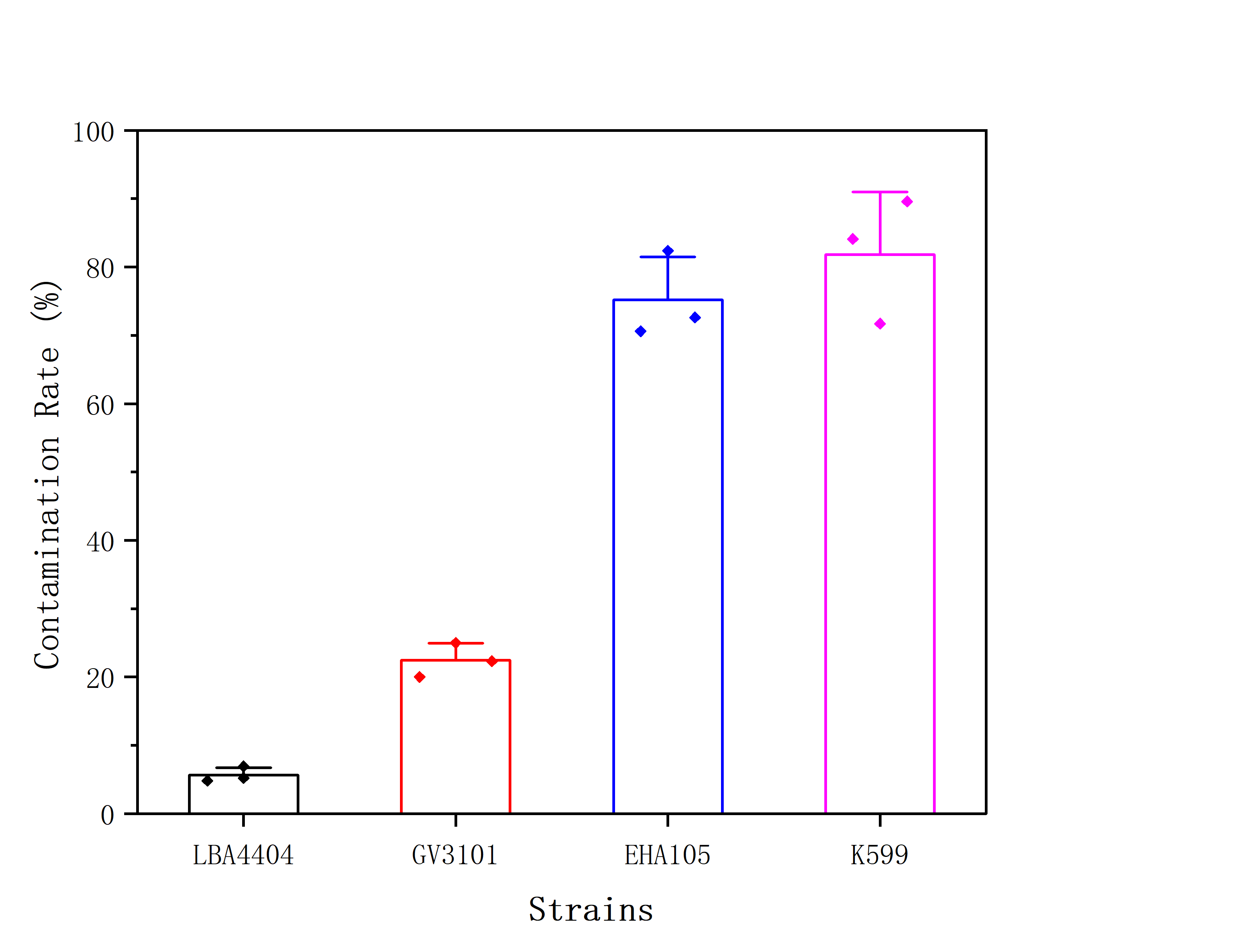
**

**Fig. S3** The leaf disk contamination rates in different *Agrobacterium* strains within two weeks. Note: contamination rate calculated as: (number of contaminated leaf disk /total number of treated leaf disks) × 100%.


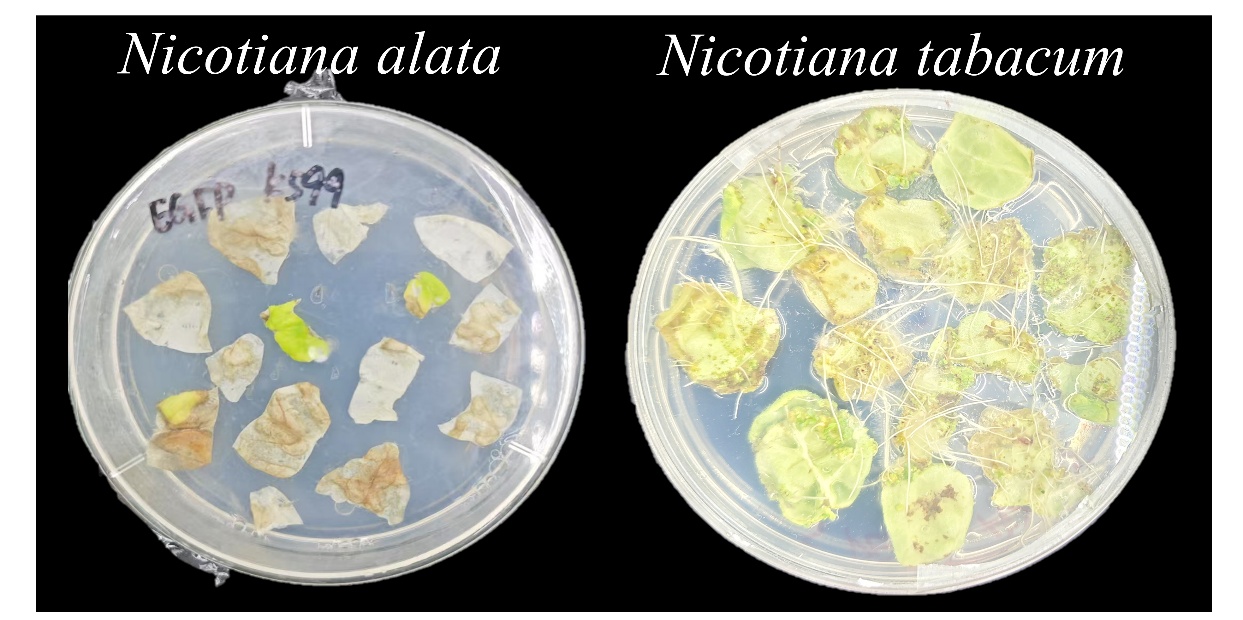


**Fig. S4** Comparison of *N. alata* (left) and *Nicotiana tabacum.L* (right) explants infected with *R. rhizogenes* K599.


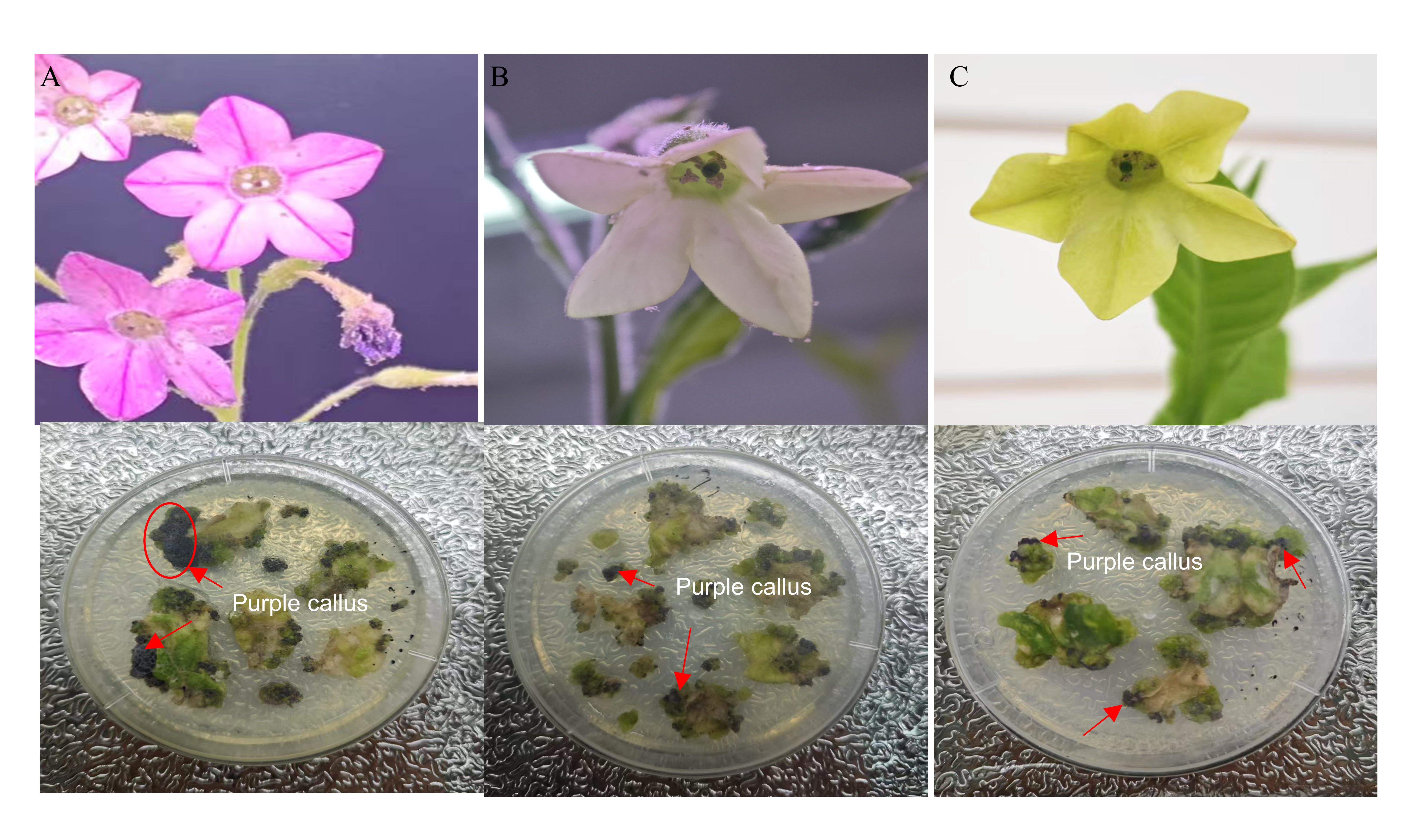


**Fig. S5** Callus phenotypes of *AtPAP1*-transgenic *N. alata* with different flower colors. **A** Pink-flowered; **B** White-flowered; **C** Lemon green-flowered.


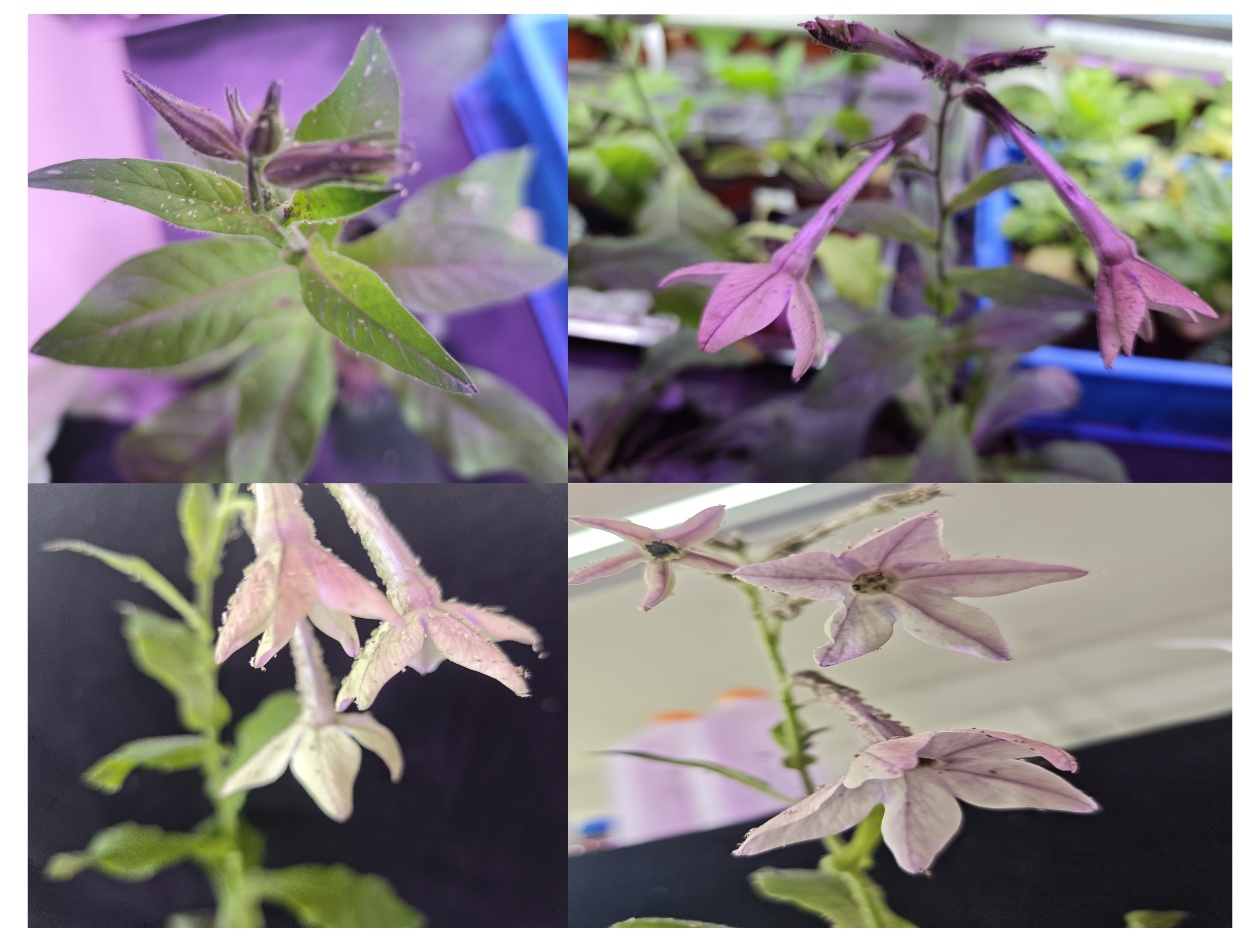


**Fig. S6** Phenotypes of *N. alata* transgenic lines overexpressing *AtPAP1*.

**Fig. S7.** Phenotypic observation of *NtAN2* transgenic *N. alata*. **A** Process of *NtAN2* transgenic *N. alata*. **B** Different *NtAN2* transgenic *N. alata* plants.


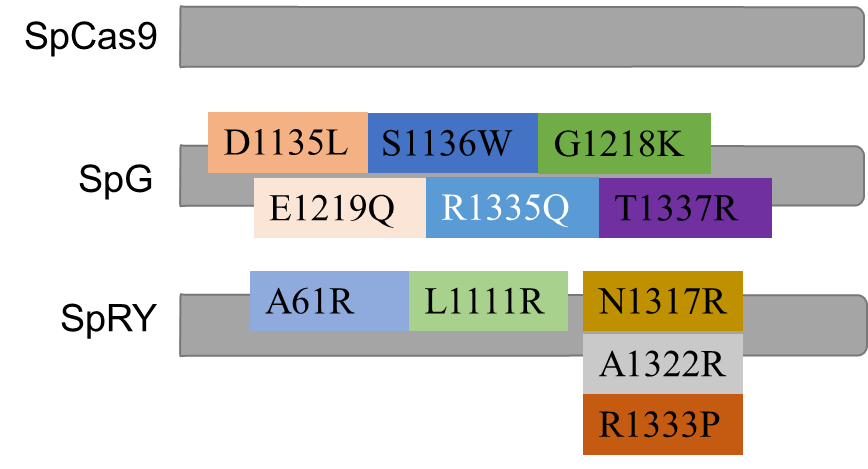


**Fig. S8.** Schematic of SpCas9 and its variants SpG and SpRY.


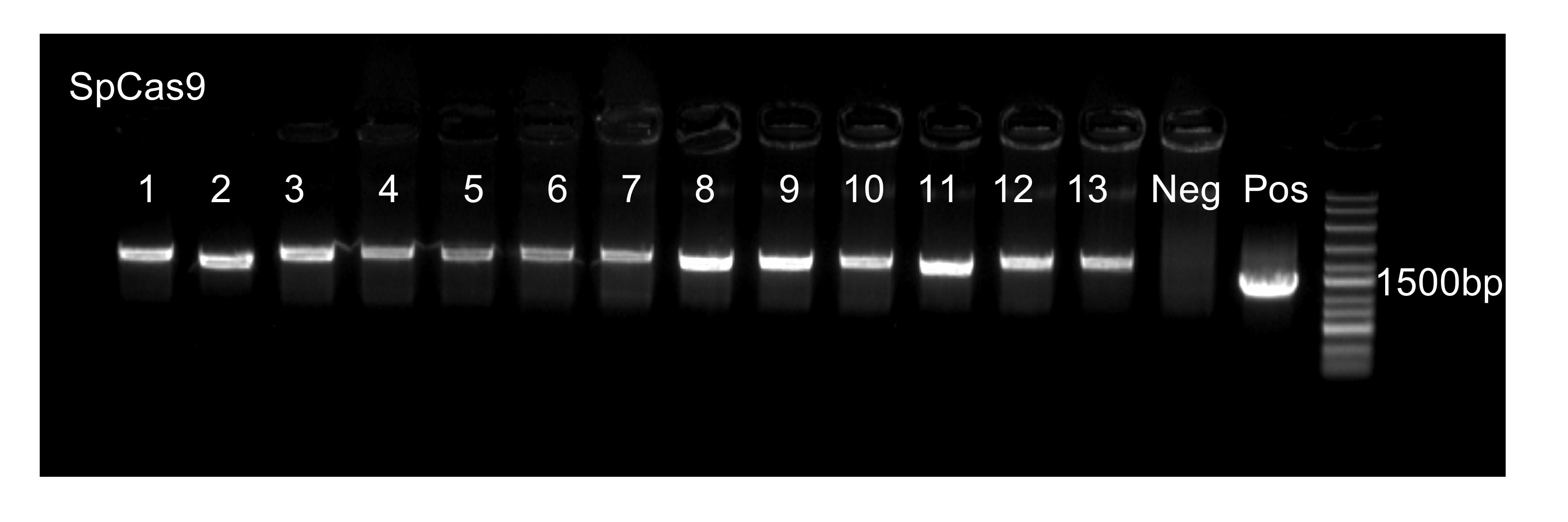


**Fig. S9.** PCR verification (length: 1613bp) results of SpCas9 integration in randomly selected *PDS* edited lines of *N. alata*. “Neg” is the negative control, and “Pos” is the positive control.


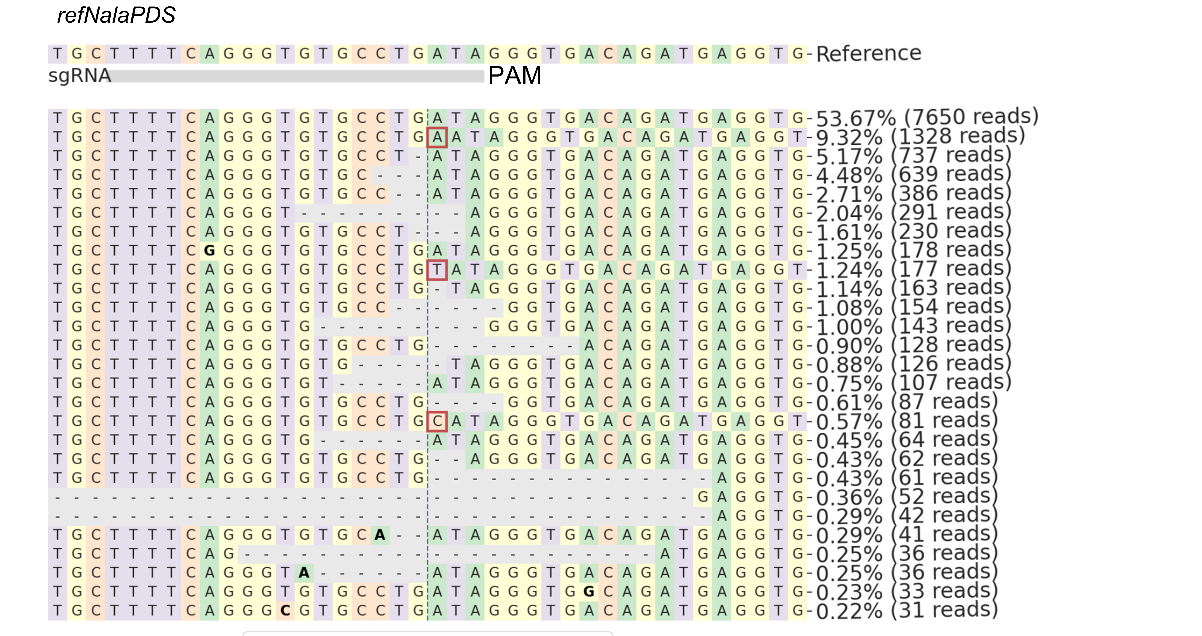


**Fig. S10.** NGS results of SpCas9-mediated editing at the *N. alata* PDS gene.


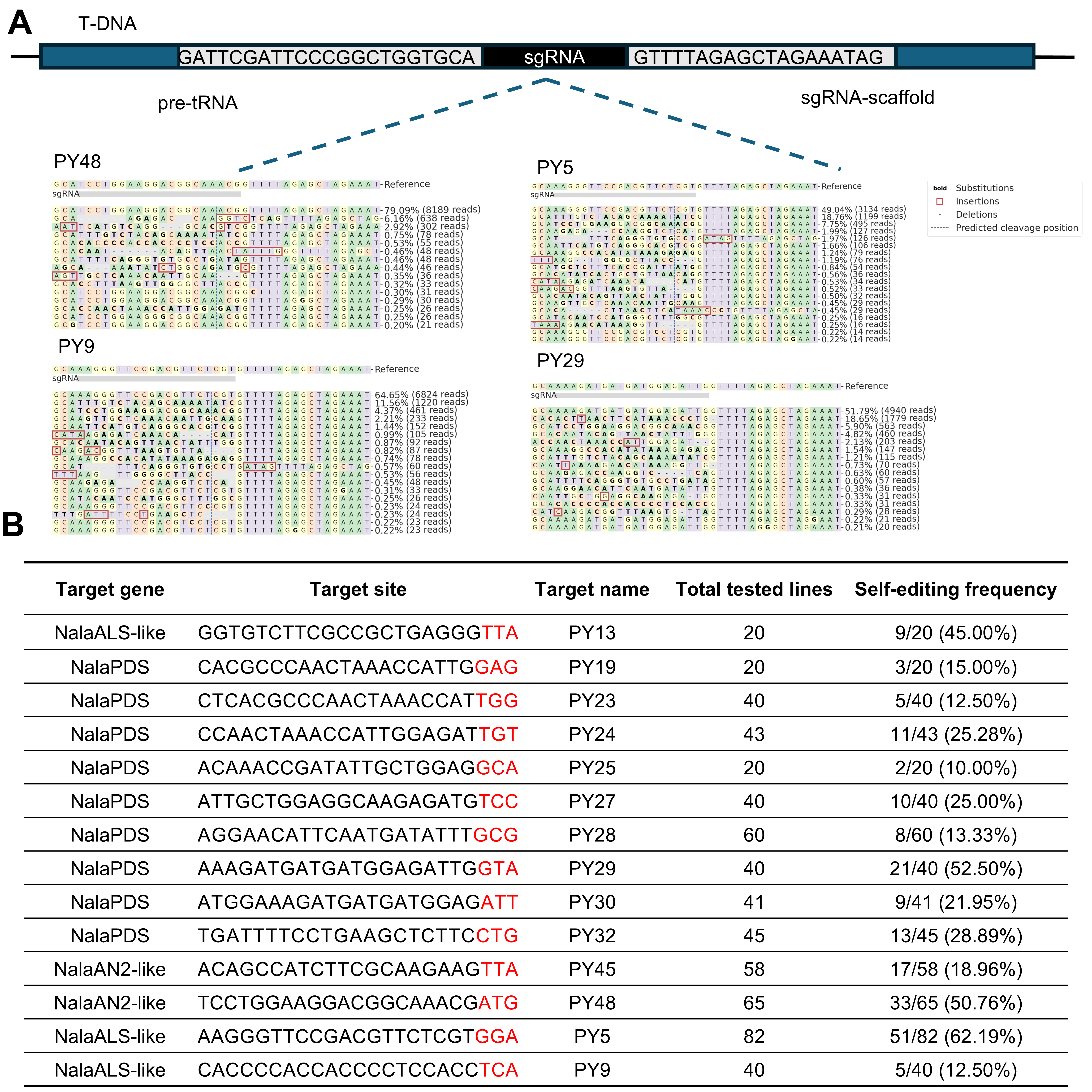


**Fig. S11.** Sequence characteristics and frequency statistics of self-editing events in SpRY-mediated gene editing. **A** High-throughput sequencing profiles of target regions in representative edited lines (PY48, PY5, PY9, PY29). **B** Statistical table self-editing frequencies across different targets.


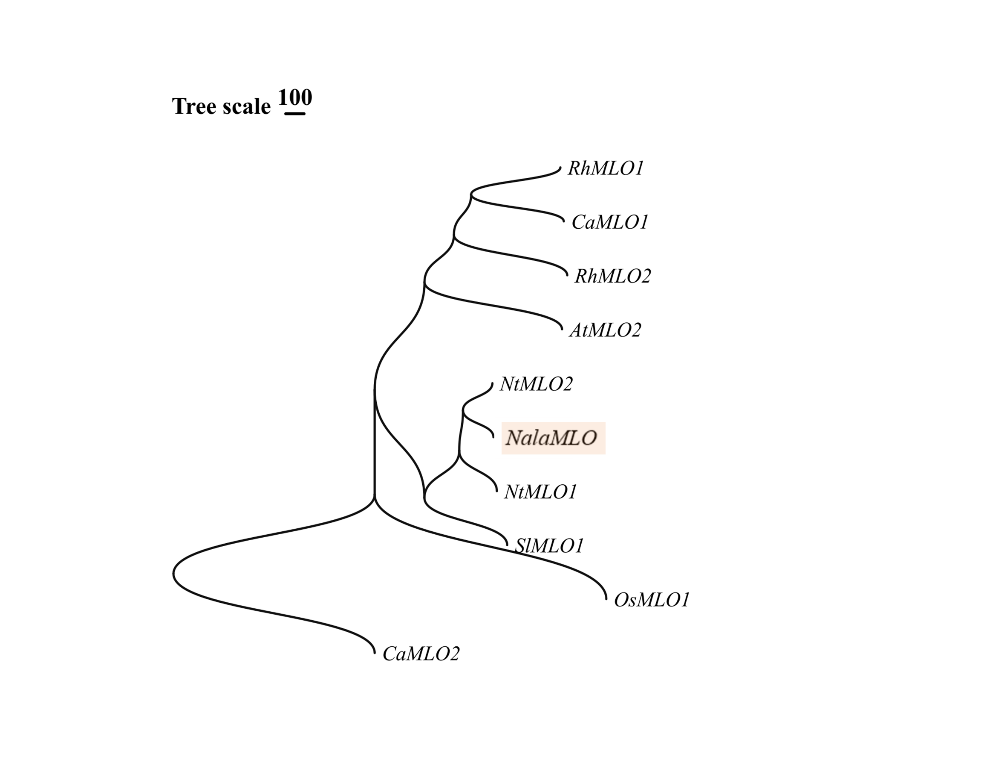


**Fig. S12.** Phylogenetic tree of *NalaMLO* gene. The neighbor-joining phylogenetic tree was constructed based on the amino acid sequences of MLO proteins, including those from *N. alata* (*NalaMLO*, highlighted), *Nicotiana tabacum* (*NtMLO1*, *NtMLO2*), *Arabidopsis thaliana* (*AtMLO2*), *Solanum lycopersicum* (*SlMLO1*), *Oryza sativa* (*OsMLO1*), *Capsicum annuum* (*CaMLO1*, *CaMLO2*), and *Rosa hybrida* (*RhMLO1*, *RhMLO2*). The tree scale represents evolutionary distance.

**
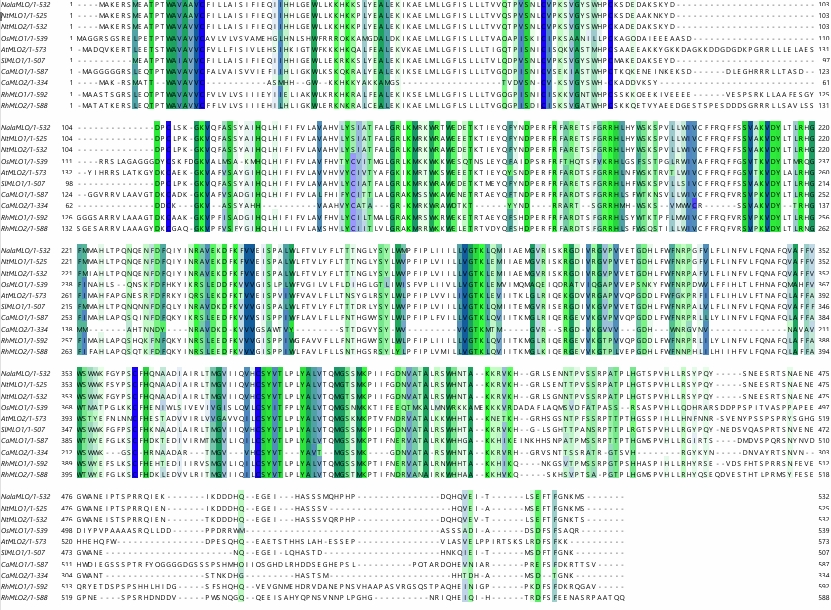
**

**Fig. S13.** Multiple sequence alignment of MLO protein sequences from various plant species.


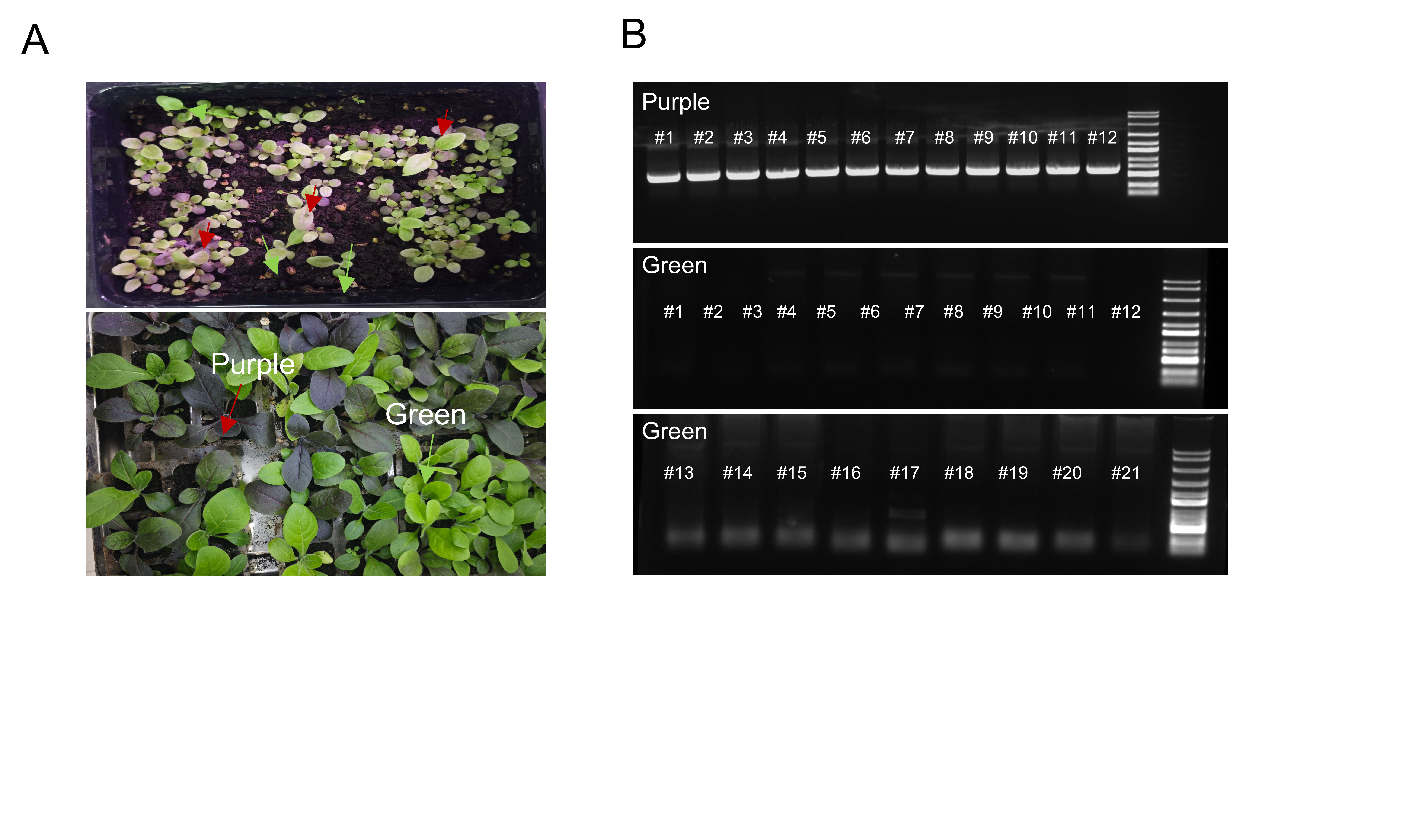


**Fig. S14.** Screening for transgene-free *MLO*-edited T_1_ plants. **A** Phenotypes of T_1_ seedlings derived from T_0_ plant (#2), showing purple and green phenotypes. **B** PCR detection results of purple seedlings and green seedlings using T-DNA primers. All green T_1_ progeny were free of transgenic elements.


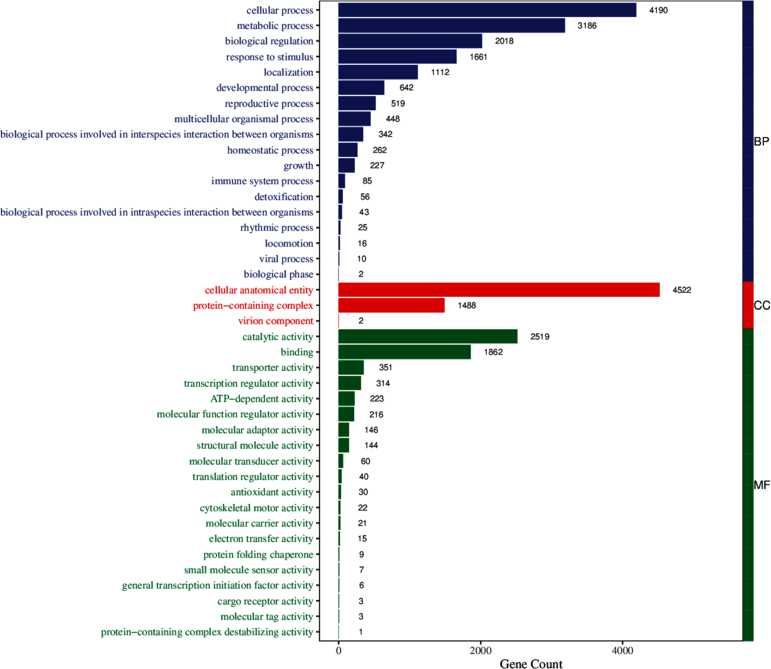


**Fig. S15.** Bar chart of Gene Ontology (GO) analysis for transcripts, categorizing terms into Biological Process (BP, blue), Cellular Component (CC, red), and Molecular Function (MF, green). The x-axis shows gene counts, and the y-axis lists specific GO terms.





**Fig. S1****6.** Metabolomic analysis of differentially accumulated metabolites (DAMs) between wild-type (WT) and *Nalamlo* lines. **A** Volcano plot depicting DAMs. Each dot represents a metabolite; red dots indicate significantly up-regulated metabolites, blue dots indicate significantly down-regulated metabolites, and gray dots indicate metabolites with no significant difference (screening criteria: |log₂(fold change)| > 1, *P* < 0.05). **B** KEGG pathway enrichment analysis of DAMs, illustrating the percentage distribution of DAMs across major metabolic pathways. **C** Heatmap showing the top 10 most significantly up-regulated and down-regulated DAMs between WT and *Nalamlo* lines, where red indicates higher metabolite accumulatiand blue indicates lower accumulation.
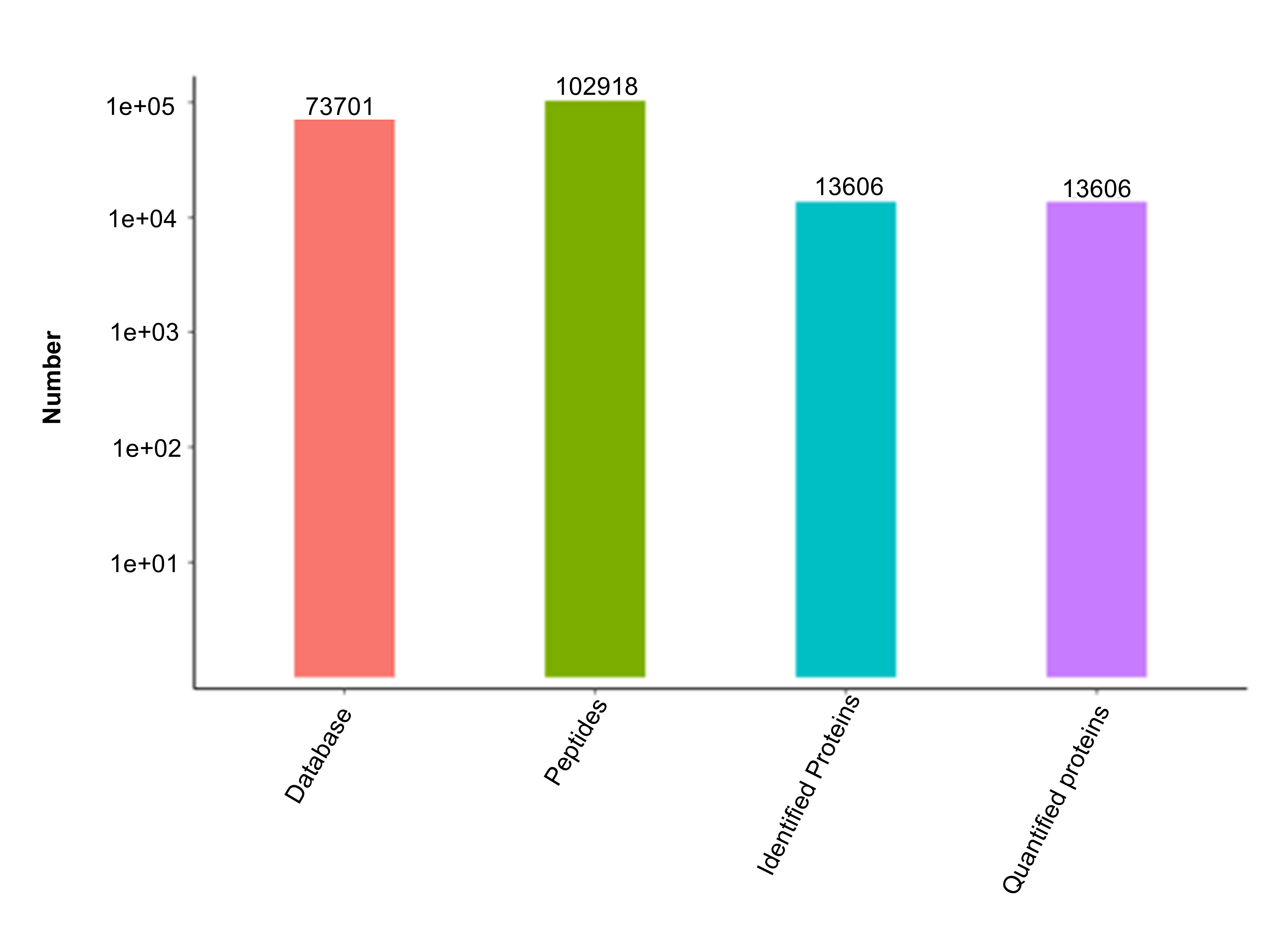


**Fig. S17.** Summary of mass spectrometry-based proteomic profiling. This bar chart displays key metrics from the proteomic analysis, including the number of database entries searched (73,701), unique peptides identified (102,918), total proteins identified (13,606), and successfully quantified proteins (13,606). The y-axis employs a logarithmic scale to represent the count for each category.


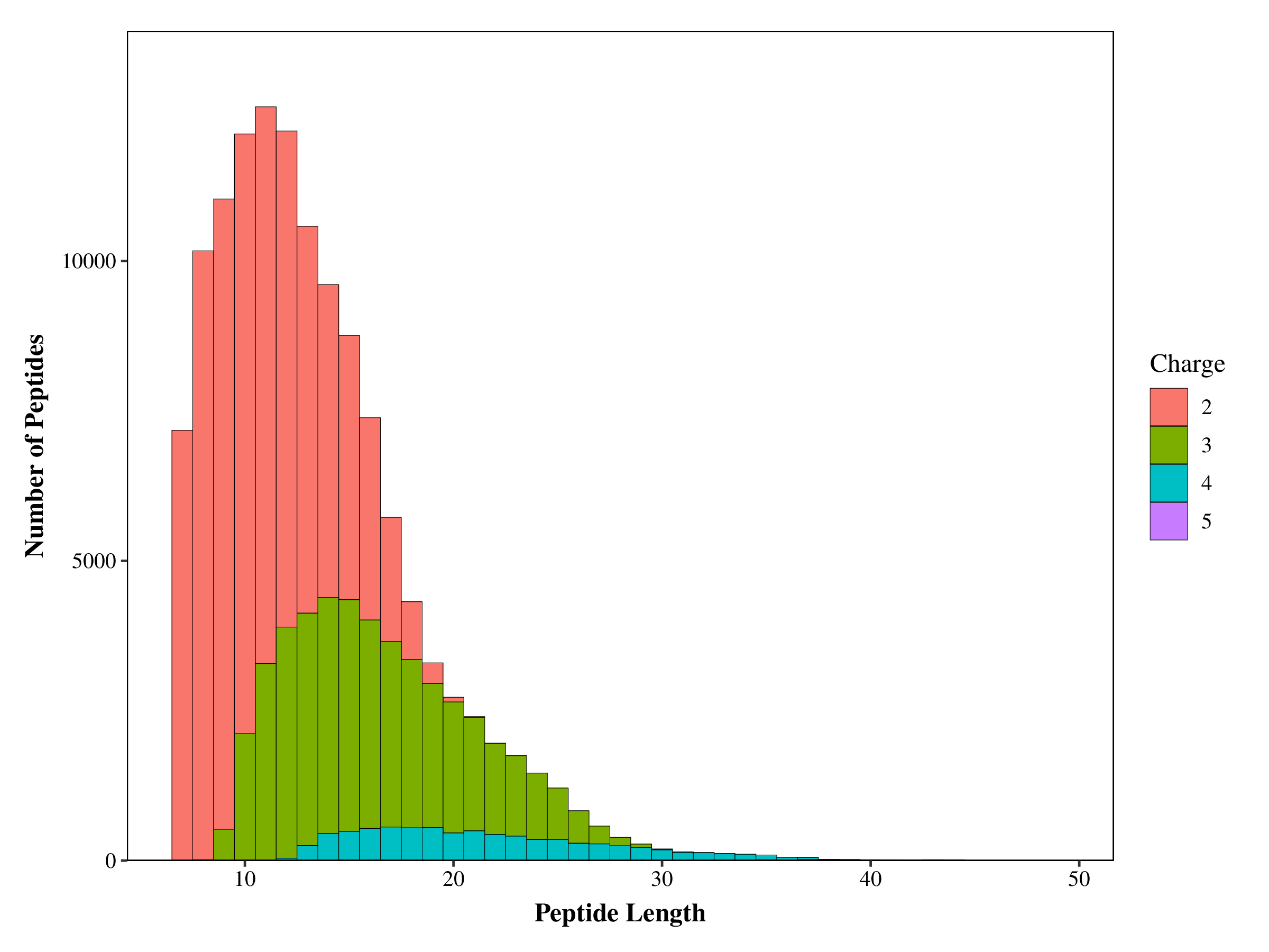


**Fig. S18.** Distribution of peptide length and charge in proteomic analysis. This histogram depicts the number of identified peptides as a function of peptide length (x-axis) and charge state (color-coded: charge 2 in red, charge 3 in green, charge 4 in cyan, charge 5 in purple). Most peptides range in length from ~8 to 20 amino acids, with peptides carrying a charge of 2 being the most abundant, which is consistent with typical features of peptides detected by mass spectrometry.


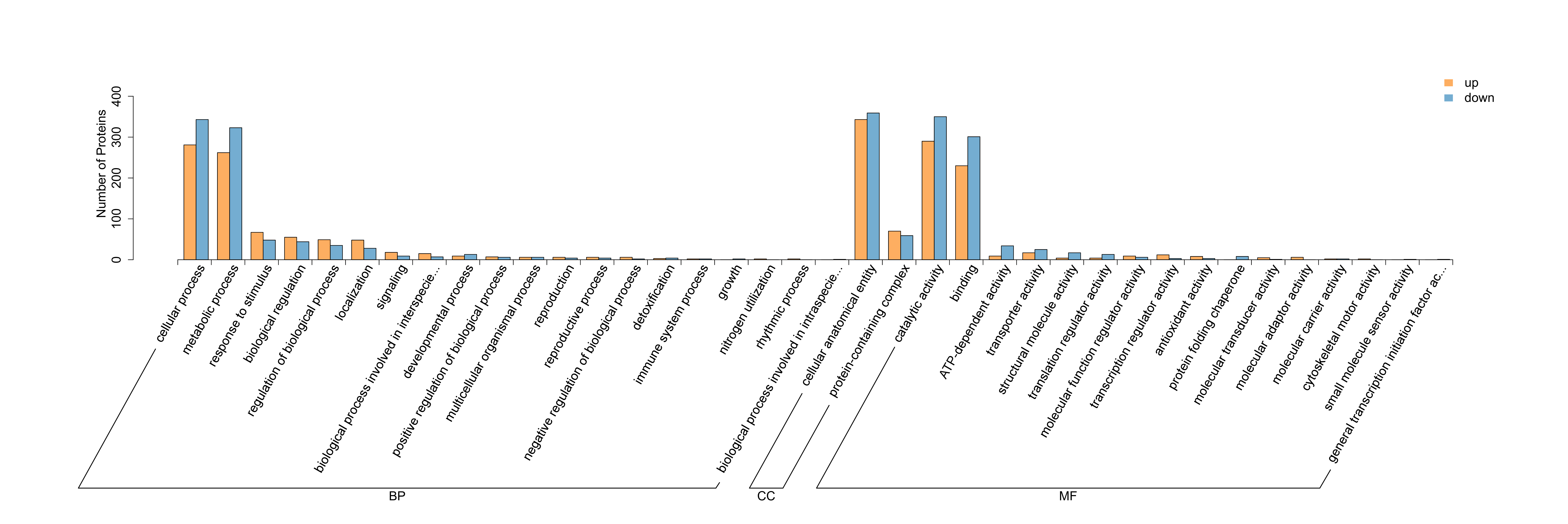


**Fig. S19.** Gene Ontology (GO) enrichment analysis of differentially abundant proteins (DAPs). The histogram illustrates the number of up-regulated (UP, orange) and down-regulated (down, blue) DEPs across different GO terms, which are classified into three categories: biological process (BP), cellular component (CC), and molecular function (MF).
